# Origin and evolution of West Nile virus lineage 1 in Italy

**DOI:** 10.1017/S0950268824001420

**Published:** 2024-12-02

**Authors:** Andrea Silverj, Giulia Mencattelli, Federica Monaco, Federica Iapaolo, Liana Teodori, Alessandra Leone, Andrea Polci, Valentina Curini, Marco Di Domenico, Barbara Secondini, Valeria Di Lollo, Massimo Ancora, Annapia Di Gennaro, Daniela Morelli, Maria Gabriella Perrotta, Giovanni Marini, Roberto Rosà, Nicola Segata, Omar Rota-Stabelli, Annapaola Rizzoli, Giovanni Savini, West Nile Virus Working Group

**Affiliations:** 1 Centre Agriculture Food Environment, University of Trento, San Michele all’Adige, Trento, Italy; 2 Research and Innovation Centre, Fondazione Edmund Mach, San Michele all’Adige, Trento, Italy; 3 Department CIBIO, University of Trento, Trento, Italy; 4 Istituto Zooprofilattico Sperimentale dell’Abruzzo e del Molise, Teramo, Italy; 5 Centro Nazionale di Lotta ed Emergenza Contro le Malattie Animali, Rome, Italy; 6 National Biodiversity Future Center (NBFC), Palermo, Italy; 7 The members of the West Nile virus working group are listed in the group authorship list, located in supplementary materials

**Keywords:** West Nile virus, lineage 1, Europe, Italy, phylogeography, mosquito

## Abstract

West Nile virus (WNV) is a mosquito-borne pathogen that can infect humans, equids, and many bird species, posing a threat to their health. It consists of eight lineages, with Lineage 1 (L1) and Lineage 2 (L2) being the most prevalent and pathogenic. Italy is one of the hardest-hit European nations, with 330 neurological cases and 37 fatalities in humans in the 2021–2022 season, in which the L1 re-emerged after several years of low circulation. We assembled a database comprising all publicly available WNV genomes, along with 31 new Italian strains of WNV L1 sequenced in this study, to trace their evolutionary history using phylodynamics and phylogeography. Our analysis suggests that WNV L1 may have initially entered Italy from Northern Africa around 1985 and indicates a connection between European and Western Mediterranean countries, with two distinct strains circulating within Italy. Furthermore, we identified new genetic mutations that are typical of the Italian strains and that can be tested in future studies to assess their pathogenicity. Our research clarifies the dynamics of WNV L1 in Italy, provides a comprehensive dataset of genome sequences for future reference, and underscores the critical need for continuous and coordinated surveillance efforts between Europe and Africa.

## Introduction

West Nile virus (WNV) is an arthropod-borne flavivirus member of a Japanese encephalitis serocomplex within the *Flaviviridae* family. In nature, it is maintained by several species of birds and competent mosquitoes mainly belonging to the *Culex* species. The former generally act as amplifying hosts, while the latter as vectors [1, 2]. Humans and other animals are considered incidental dead-end hosts. In humans, most of the infections are mild (flu-like symptoms) or asymptomatic (West Nile fever, WNF) [3]. In some cases (less than 1%), however, especially in older or immunocompromised people, they can cause severe and sometimes fatal neurological disease (WNND) [3].

Since its first identification in Uganda in 1937 [4], WNV has progressively spread to Europe, America, Asia, and Oceania, becoming the most widespread arbovirus in the world [5]. Eight phylogenetic lineages are currently known: Lineage 1 (L1) to Lineage 8 (L8). Of them, WNV L1 and L2 are by far the most widespread and pathogenic. They are often associated with severe outbreaks in humans, horses, and birds [6]. WNV L1 cases started to be recorded in Europe in the middle 1950s [7], while phylogenetic studies show that the virus arrived in North America from the Middle East (Israel) in the 1990s (where it was first detected in 1999, but probably it had emerged the year before) [8–10]. These and many of the subsequent introductions could be attributed to migratory birds or result from long-distance transport of infectious animals related to international trade [11, 12]. The circulation of WNV L1 has been observed in several European countries, and major clades have been characterized: (i) the Western Mediterranean clade, with strains first identified in Italy (1998) and France (2000) and (ii) the Eastern European clade that includes strains responsible for the outbreaks in Romania (1996) and Russia (1999) [10, 13, 14]. The 1998 WNV Italian strain was reported in the Tuscany region. It was able to cause severe neurological disease and deaths in horses, but no cases were recorded in humans [15]. A re-emergence of WNV L1 strains belonging to the Western Mediterranean clade was reported 10 years later in North-Eastern Italian regions (Emilia-Romagna, Veneto, and Lombardy). They were responsible for severe illness in humans and horses. Numerous birds and mosquitoes were also found infected with WNV L1 [16] (https://westnile.izs.it/j6_wnd/wndItalia). In 2010, the circulation of WNV L1 was also observed in Southern Italy, and the outbreaks were reported in Sardinia in 2011 [17, 18]. In the same year, the circulation of WNV L2 was detected for the first time in the Italian territory. It soon became the most prevalent lineage circulating in the country [19]; meanwhile, WNV L1 has been more and more sporadically reported. Evidence of its circulation was, however, observed in North-Eastern regions (2012–2014, 2017), Sardinia (2015–2016), and Campania (2020) [14].

In 2021, unexpectedly, a new WNV L1 incursion re-occurred in the Northern part of Italy (https://westnile.izs.it/j6_wnd/docBolletItaPeriodico?annoDocumento=2021). The virus re-emerged in the same area in 2022, co-circulating with WNV L2 and causing nearly 300 WNND cases and 37 deaths in humans. WNV positivities were also reported in 45 horses and 349 wild and target birds. Similarly, numerous infected mosquito pools were found (https://westnile.izs.it/j6_wnd/docBolletItaPeriodico?annoDocumento=2022). The Veneto region was the major hotspot area for viral circulation, with 142 human WNND and 17 deaths [20], (https://westnile.izs.it/j6_wnd/docBolletItaPeriodico?annoDocumento=2022).

The reappearance of WNV L1 in Italy, the pathogenic differences of the various circulating strains, the increasing incidence of WNND cases in humans, together with the gap of knowledge of several aspects of WNV biology, ecology, and genomics along with the lack of safe vaccines and specific therapies provide the motivation to improve research and deepen the understanding of the genetic features of the WNV L1 strains circulating in Italy. To this end, in this study, we have integrated a new genome dataset of WNV L1 strains circulating in Italy between 2008 and 2022 with a worldwide dataset of WNV L1 sequences publicly available in the NCBI. Furthermore, we have combined this consistent dataset with phylogenetic and phylogeographic analyses of L1 strains, displaying the dynamics of the viral circulation in the Italian territory and uncovering the spatial and temporal patterns of the virus between Western-Mediterranean countries.

## Methods

### Sample collection

WNV L1-positive samples were collected in Italy between 2008 and 2022 within the national surveillance plan, coordinated by the Ministry of Health, the Istituto Superiore di Sanità (epidemiology and national reference laboratory, human), and the Istituto Zooprofilattico of Abruzzo and Molise (IZS-Teramo) (epidemiology and national reference laboratory, animal/entomology) (https://westnile.izs.it/j6_wnd/home, https://www.epicentro.iss.it/westnile/). The plan is based on an integrated surveillance on birds, mosquitoes, horses, and humans, conducted on a regional scale in Italy [21]. Any positive results from local surveillance activities are confirmed by the National Reference Centre for Foreign Animal Diseases (CESME) at IZS-Teramo. The official veterinary authorities are in charge of registering any notifications in the national information system for the notification of outbreaks in animals (SIMAN) [22].

### Sample analysis

#### Tissue homogenization, RNA extraction, real-time RT-PCR

At IZS-Teramo, pools of mosquitoes, bird organs (heart, kidney, spleen, and brain), collected from either residential or wild birds, and horse samples (blood or brain) were homogenized in phosphate-buffered saline (PBS) with antibiotics. Viral RNA was extracted by using the MagMAX CORE Nucleic Acid Purification KIT (Applied Biosystem; Thermo Fisher Scientific, Life Technologies Corporation, TX, USA) according to the manufacturer’s instructions. The extracted viral RNA was amplified using two real-time reverse transcription polymerase chain reactions (qRT-PCR), one aiming for the simultaneous detection of WNV L1 and L2 [23], and the other aiming for the detection of all WNV lineages [24]. For Usutu virus, the qRT-PCR was performed as previously described by Cavrini et al. [25].

### Sequence data preparation and retrieval

WNV-L1 positive samples were selected for Illumina and Sanger sequencing. Particularly, purified nucleic acids extracted from the WNV L1 viral strains obtained from samples collected in Italy between 2008 and 2012 were sequenced by using the Sanger methods [26]. Briefly, the total RNA was extracted from the collected sample using a High Pure Viral Nucleic Acid Kit (Roche Diagnostics GmbH; Roche Applied Science, Mannheim, Germany), according to the manufacturer instructions, and collected in 45 μL elution buffer prewarmed at 72 °C. The complete WNV-coding DNA sequences (cds) of the polyprotein precursor gene were amplified using 13 WNV primer pairs able to amplify 13 overlapping regions of the genome (the primer sequences can be found in Additional file 1: Table S1). Gel-based RT-PCR was performed using a Transcriptor One-Step RT-PCR kit (Roche Diagnostics Deutschland GmbH, Mannheim, Germany) according to the manufacturer’s instructions. The RT-PCR cycling conditions for the amplification were 50°C for 15 min and 94°C for 7 min, followed by 35 cycles of denaturation at 94°C for 10 s, annealing at 57.5°C for 30 s, and extension at 68°C for 4 min and 30 s, followed by 1 extension cycle performed at 68°C for 7 min. The gel-based RT-PCR amplicons were purified with a Qiaquick PCR Purification kit (Qiagen, Leipzig, Germany). The purified amplicons and the 13 WNV sequencing primers were sent to an external service, Eurofins Genomics (Eurofins Genomics, Germany GmbH, Ebersberg, Germany), to perform sequencing in both directions. The obtained sequences were analysed with SeqScape v3.0 (Thermo Fisher Scientific, Waltham, MA, USA). Furthermore, purified nucleic acids, obtained from the WNV L1-positive samples collected in Italy between 2020 and 2022, were sequenced by next-generation sequencing. In detail, each positive sample total RNA was subjected to Turbo DNase treatment (Thermo Fisher Scientific, Waltham, MA, USA) at 37°C for 20 min and then purified with an RNA Clean and Concentrator-5 Kit (Zymo Research, Irvine, CA, USA). The purified RNA was used for the assessment of the sequence-independent single primer amplification (SISPA) protocol [14, 27]. Following the SISPA protocol, a single-strand cDNA was obtained using reverse transcription (RT) in 20 μL reaction mixture with 5X SSIV buffer, 50 μM random hexamer FR26RV-N 50-GCCGGAGCTCTGCAGATATCNNNNNN-30, 10 mM dNTPs mix, 100 mM DTT, 200 units SuperScript IV Reverse Transcriptase (Thermo Fisher Scientific, Waltham, MA, USA), and 40 U RNAse OUT RNase inhibitor (Thermo Fisher Scientific, Waltham, MA, USA) following the manufacturers’ instructions. The reaction was incubated at 23°C for 10 min, 50°C for 50 min, and 80°C for 10 min. To convert the single-stranded cDNA into double-stranded (ds) cDNA, 1 μL (2.5 U) 3′-5′ Klenow Polymerase (New England Biolabs, Ipswich, MA, USA) was directly added to the reaction. The incubation was carried out at 37°C for 1 h and 75 °C for 10 min. Next, 5 μL of ds cDNA was amplified with a PCR master mix containing 5× Q5 reaction buffer, 10 mM dNTPs, 40 μM random primer FR20 Rv 50 -GCCGGAGCTCTGCAGATATC-30, 0.01 U/μL Q5 High Fidelity DNA polymerase (NEB, New England Biolabs, Ipswich, MA, USA), and 5× Q5 High Enhancer. The incubation was performed with the following thermal conditions: 98°C for 1 min, 40 cycles of 98°C for 10 s, 65°C for 30 s and 72°C for 3 min, and a final extension step of 72°C for 2 min. The PCR product was purified using Expin TM PCR SV (GeneAll Biotechnology CO., Seoul, Korea) and then quantified using a Qubit DNA HS Assay Kit (Thermo Fisher Scientific, Waltham, MA, USA). The sample was diluted to obtain a concentration of 100–500 ng, then used for library preparation with an Illumina DNA prep kit, and sequenced with a NextSeq 500 (Illumina Inc., San Diego, CA, USA) using a NextSeq 500/550 Mid Output Reagent Cartridge v2, 300 cycles, and standard 150 bp (base pairs) paired-end reads. After quality control with FastQC tool v0.11.5 (Bioinformatics Group, Babraham Institute, Cambridge, UK) [28, 29] and trimming with Trimmomatic v0.36 (Usadellab, Düsseldorf, Germany) [30], raw reads (available upon request to the corresponding authors) were *de novo* assembled using SPADES v3.11.1 (Algorithmic Biology Lab, St Petersburg, Russia) [31]. The contigs obtained were analysed with BLASTn to identify the best match reference. We introduced snippy as aligner (version 4.5.1, default parameters; https://github.com/tseemann/snippy) and iVar (version 1.3.1, command consensus, -q 20, -m 1) to obtain consensus sequences [32]. This procedure was also integrated in a pipeline for the West Nile lineage prediction on GENPAT platform (https://genpat.izs.it/cmdbuild/ui/#login) of the “National Reference Centre for Whole Genome Sequencing of microbial pathogens: database and bioinformatic analysis.” Thirty-one WNV L1 consensus sequences were obtained at IZS-Teramo after trimmed reads were mapped to the WNV L1 reference sequence FJ483548 (Italy, 2008).

Metadata for the IZS-Teramo newly generated sequences were obtained from the Laboratory Information Management Systems (SILAB) at IZS-Teramo (https://www.izs.it/IZS/Engine/RAServePG.php/P/257610010719/L/1). A worldwide dataset of 130 WNV L1 sequences have been downloaded from the Supplementary Materials of [21], including sequences obtained from NCBI on 01/03/2022. By using a custom R script for automatic sequence retrieval, a new search has been conducted on NCBI on 06/11/2022 and 28 newly published WNV L1 sequences >= 200 nt have been downloaded.

### Sequence data cleaning and formatting

All sequences were quality-filtered and only data for sequences longer than 10 kb (i.e., sequences covering almost the entire WNV genome) were retained for subsequent steps, as described elsewhere [21]. After this step, 2 sequences (OP734273, OU953898.1) were removed, as they contained a percentage of ambiguous bases above 10%. A total of 186 WNV L1 genomes, 55 of which coming from Italy (30 of which belonging to the IZS newly obtained dataset), were selected for further analysis. A table of sequence curated metadata can be found in Additional file 2: Table S2.

### Alignment, recombination detection, and model selection

All the datasets were aligned individually using MAFFT v7 (https://mafft.cbrc.jp/alignment/server/) with the “--auto” option, and trimmed using trimAl v2 with the “-automated1” option [33]. The presence of recombinant sequences in the final dataset was checked by running the RDP4 program [34], with default options. The sequence AJ965626.2 from Portugal was indicated as suspect recombinant and was therefore excluded from the alignment. Model selection was carried out on all datasets using ModelFinder [35], implemented in IQTREE2 [36], using parameters “-T AUTO -m TESTONLY.” The best-fit model for both the worldwide and the Bayesian down-sampled datasets was GTR+F+I+G4, chosen according to both Akaike Information Criterion (AIC) and Bayesian Information Criterion (BIC).

### Maximum likelihood phylogenies

A maximum likelihood phylogeny of the dataset including the 185 WNV L1 sequences was reconstructed by using RAxML v8.2.12 [37], with commands “-p 1989 -m GTRGAMMAI -x 2483 -# 100 -f a -T 20”. Clades were annotated using the resulting topology when having bootstrap supports ≥90.

### Molecular clock and phylogeographic analysis

We used the results obtained in the maximum likelihood analysis to subset our dataset for molecular clock analysis. In particular, we selected a highly supported group (see Figure 2), which includes clades 1–4 and all Italian genomes.

A full list of the sequence identifiers and the associated meta-data (retrieved from the NCBI or the literature) is provided in the Additional file 3: Table S3. When the exact position was not available, we approximated the location using the coordinates of the municipality from which the sample was collected. Phylogeography was reconstructed by using continuous traits (latitudinal and longitudinal coordinates for each sequence) in BEAST v1.10.4 [38]. We divided the analysis into two different partitions (one for sequence data and the other for continuous coordinates) and used the relaxed random walk Cauchy diffusion model for our location partition, with bivariate traits representing latitude and longitude, adding random jitter to the tips (jitter window size: 0.01). For the location partition, we selected the option to reconstruct states at all ancestors. We employed an uncorrelated relaxed clock with a log-normal distribution and a Coalescent Bayesian Skyline tree prior. We ran four different analyses, setting for each run an MCMC length of 200 × 10^6^ generations, sampling every 20000 steps. Convergence was assessed using Tracer v1.7.1 [39], to check the effective sample size (ESS) of each independent run and of the combined results, setting the burn-in values and making sure that all parameters were above a significance threshold of ESS (>200) for the combined run. We combined the four independent tree files using LogCombiner [38], setting the burn-in percentages as follows: run1 = 30%, run2 = 50%, run3 = 45%, run4 = 45%. A maximum-clade credibility tree using median-heights was obtained with TreeAnnotator [38], using the combined file with 460 × 10^6^ states obtained with LogCombiner after the burn-in. Phylogenetic trees and phylogeographic diffusion patterns were represented using custom R scripts, employing the ggtree [40] and SERAPHIM [41] packages, respectively. A sensitivity analysis to check for the robustness of our results to differences in sampling was carried out by generating three datasets by randomly subsampling the dataset used for phylogeographic analysis with a custom Python script (available at https://github.com/andrea-silverj/WNV-Afr_Eur/tree/main/scripts/sequence_random-sampling). Specifically, we obtained 75% (52 genomes), 50% (35 genomes), and 30% (21 genomes) reduced datasets that were analysed with the same methods and models as described previously. To reach convergence (ESS > 200), we ran the Bayesian analyses setting the following parameters: 30% dataset: MCMC length of 250 × 10^6^ generations, sampling every 25000 steps; 50% dataset: 8 runs with MCMC length of 500 × 10^6^ generations, sampling every 50000 steps, that were then combined together; 75% dataset: MCMC length of 500 × 10^6^ generations, sampling every 50000 steps. In all cases, we set a 10% burn-in.

### Full-length polyprotein analysis

Using Ugene software v44.0 (https://ugene.net/download.html), a sequence comparison between the amino acid residues of the group of strains included in the phylogeographic analysis was performed. In particular, we compared the 2021–2022 group of Italian sequences and the strains: (i) OP850023, Italy, Campania region, 2022; (ii) MW627239, Italy, Campania region, 2020; (iii) Italy, 2008 group; (iv) Italy, 2011 group; (v) AF404757, Italy, Tuscany region, 1998; (vi) MT863559, France, 2015; (vii) OU953897, Spain, 2020; (viii) JF719069, Spain, 2010; (ix) AY701413, Morocco, 2003; (x) AJ965628, Portugal, 2004; and (xi) DQ786573, France, 2004. We also included in the sequence comparison the strain NC_009942/1999 because, although it is not part of the major Western Mediterranean cluster analysed here, it is representative of all virulent L1 strains from the United States.

## Results

### Genome sequence analysis

**Figure 1.**
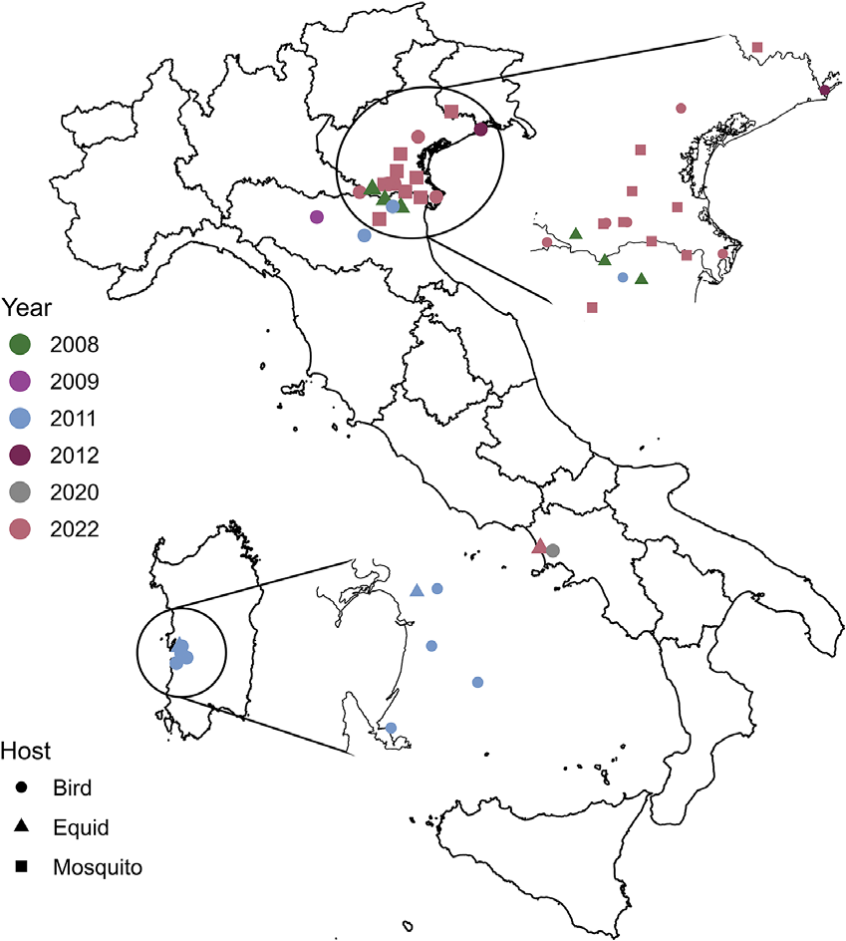
Geo-localization of West Nile virus lineage 1 sequenced sample collection sites. The sampling date is indicated by a colour scale. The shape indicates the host from which the sample was extracted. For the map, we used region border shapes from GADM (which are freely available under a CC-BY license; https://gadm.org/license.html), retrieving them through the ‘getData’ function of the ‘raster’ R package (https://www.rdocumentation.org/packages/raster/versions/3.6-20/topics/getData).

Consensus sequences were characterized by 31 WNV L1 genomes. Among them, 14 genomes were obtained from samples collected between 2008 and 2012 with Sanger sequencing, with a mean consensus length of 10263 bp. The genomes were submitted through BankIt (https://submit.ncbi.nlm.nih.gov/about/bankit/) and were released in the GenBank on 18 May 2021 under the accession numbers MW835351, MW835352, MW835353, MW835354, MW835355, MW835356, MW835357, MW835358, MW835359, MW835360, MW835361, MW835362, MW835363, and MW835364. The other 17 genomes were obtained with Illumina sequencing, with an average total number of 5096291 trimmed reads. The numbers of mapped reads (151 nucleotides [nt] in length) ranged from 159703 to 1333187, with coverage depth ranging from 2101.31× to 6381.67×. The mean consensus sequence length was 10966 bp. They were obtained from samples collected in Italy between 2020 and 2022 and were uploaded through BankIt and released in the GenBank on 26 October 2022 and 15 November 2022 under the accession numbers OP734262, OP734263, OP734264, OP734265, OP734266, OP734267, OP734268, OP734269, OP734270, OP734271, OP734272, OP734273, OP734274, OP850021, OP850022, OP850023, and MW627239. Run details for each of the 31 samples and negative controls can be found in Additional file 4: Table S4. A map of WNV L1 sequence geo-localization sites is shown in Figure 1.

### Phylogenetic tree inferred with maximum likelihood analysis

**Figure 2.**
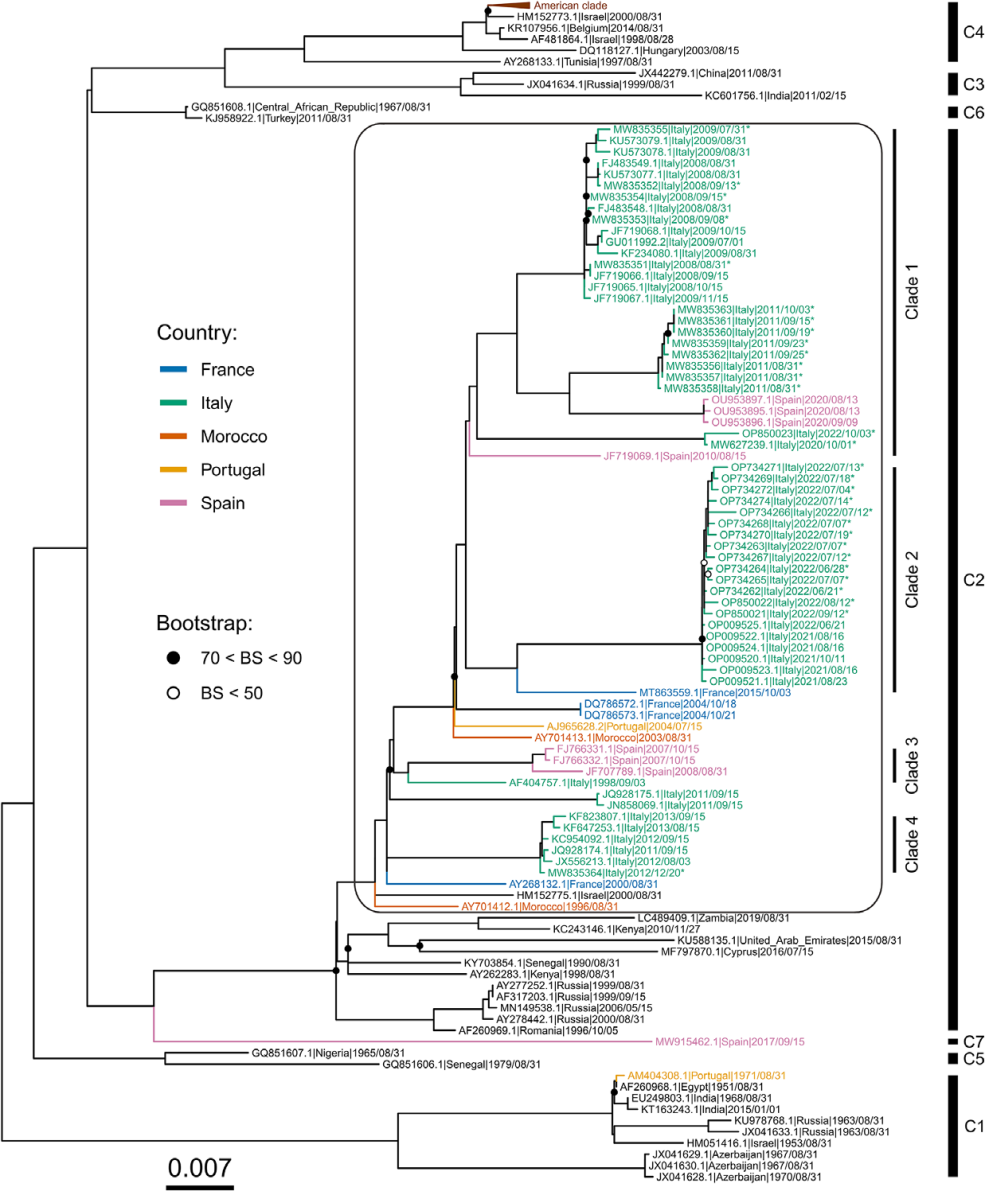
Maximum likelihood tree of West Nile virus lineage 1 sequences. Seven major clusters (C1–C7, bar on the right) can be identified. Mediterranean countries that are part of a major cluster that includes all Italian genomes (all part of the group that was used for the molecular clock and phylogeographic analysis, highlighted in a black square with rounded corners) are indicated by different colours. Inside this group, 4 Italian clades (Clade 1–4) can be defined based on the topology of the tree. Bootstrap support values (BS) >90 are not shown; bootstrap support values between 70 and 90 are represented by a full black circle; low bootstrap supports (BS < 50) are represented with a white circle. Sequences obtained in this study are indicated by the “*” symbol. A scale bar representing substitutions per site is shown at the bottom left part of the figure.

Maximum likelihood phylogenetic tree of the WNV L1 genomes analysed in this study showed the presence of seven well-supported clusters. All Italian genomes were included in cluster 2, together with a sequence of a strain from Israel (Genbank HM152775) and one from Morocco (Genbank AY701412), as shown in Figure 2.

Sequences from Italy can be divided into 4 highly supported groups (clades 1–4, Figure 2). Clade 1 includes the 2008–2009 and the 2011 groups of Italian WNV L1 sequences. They are genetically similar to the Spanish sequences of 2020 (bootstrap support = 100), and all are closely related to the 2020 (GenBank MW627239) and 2022 (GenBank OP850023) Campanian sequences (bootstrap support > 80). Clade 2 includes the 2022 WNV L1 Italian strains, originating from Veneto, Emilia-Romagna, Friuli-Venezia Giulia, and Lombardy regions (Additional file 2: Table S2). They appear to be in the same monophyletic group of the sequences obtained in the Veneto region in 2021 and closely related to the WNV L1 sequence (GenBank MT863559) obtained from a horse with neurological signs in Southern France in 2015 (bootstrap support = 100). Clade 3 includes Spanish sequences and the first WNV L1 genome ever sampled in Italy (Genbank AF404757), while clade 4 comprises sequences sampled between 2011 and 2013 near Livenza. The two remaining Italian genomes (JN858069/Italy/2011/AN-1 and JQ928175/Italy/2011/Piave), which group together, can’t be placed confidently in the tree (the signal is too low to place them with sufficient precision).

The strains DQ786572/France/2004 and DQ786573/France/2004 appear to be at the root of clade 1 and 2 (bootstrap support = 68).

The two strains collected in the Campania region in 2020 and 2022 appear to be very closely related (bootstrap support = 100) and distant from the 2021–2022 North-Eastern Italian strains.

### Phylogeographic analysis of the WNV L1 Western Mediterranean clade

**Figure 3.**
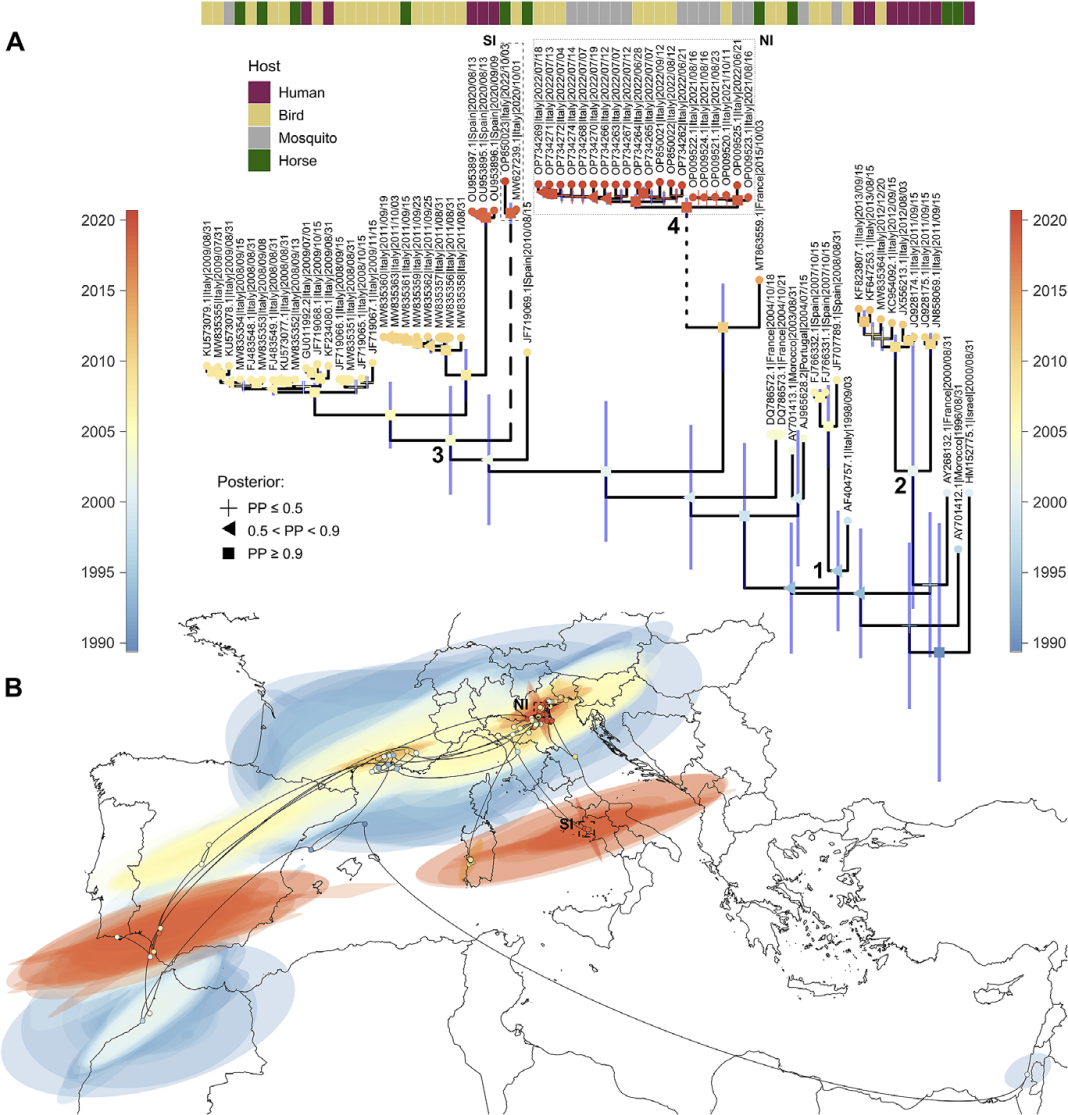
Phylogeographic analysis of the WNV L1 Western Mediterranean clade. (A) Molecular clock of the clade including all Italian sequences. Posterior probabilities of the nodes are indicated by different shapes. The bar at the top indicates the host from which the genome was isolated (human, bird, mosquito, or horse). The time scales on the right and left sides indicate, with a colour gradient, the sampling time (blue: older; red: more recent) of the genomes (see tips) or the median estimated age of their ancestors (see nodes). The four main introductions in Italy are indicated by numbers close to the nodes (1–4), while genomes from the recent outbreaks in Southern Italy (SI) and Northern Italy (NI) are highlighted by a dotted square and a full square, respectively. (B) Phylogeographic diffusion pattern in continuous scale of the virus in the Mediterranean basin. Genome sampling sites and reconstructed locations are plotted together on a map, showing the connections among all genomes and their inferred ancestors. Again, the same colour scale used in (A) indicates the sampling time of the genomes or the median estimated age of their ancestors. The coloured areas in the figure represent the 95% HPDs of the locations and times inferred by the model. For the map, we used region border shapes from GADM (which are freely available under a CC-BY license; https://gadm.org/license.html), retrieving them through the ‘getData’ function of the ‘raster’ R package (https://www.rdocumentation.org/packages/raster/versions/3.6-20/topics/getData).

We reconstructed a phylogeographic diffusion pattern for a subsample of cluster 2 (highlighted in Figure 2), including all Italian clades. We estimated the value of the evolutionary rate of the virus at a mean of 7.102 × 10^−4^ substitution/site/year (median = 7.085 × 10^−4^; 95% HPD: 4.854 × 10^−4^–9.333 × 10^−4^), which is in line with the rate of other +ssRNA viruses and with previous analyses carried out on WNV L1 [42, 43]. The position inferred for the common ancestor of the sequences suggests its initial spread in the area of the Gulf of Lion, with a possible first introduction occurring around 1989 (median = 1989.34; mean = 1987.97; 95% HPD: 1977.15–1995.48) and later appeared in the Camargue region of France around 1993 (median = 1993.53; mean = 1993.13; 95% HPD: 1988.01–1997.21). From this region, the virus moved southwards towards Morocco (AY701412.1|Morocco|1996/08/31; AY701413.1|Morocco|31 August 2003), Spain (FJ766332.1|Spain|15 October 2007; FJ766331.1|Spain|15 October 2007; JF707789.1|Spain|31 August 2008; JF719069.1|Spain|15 August 2010), and Portugal (AJ965628.2|Portugal|15 July 2004) between 1995 and 2010 and eastwards towards Israel (HM152775.1|Israel|31 August 2000) and Italy, with four introductions (see Figure 3), the first around 1998 in Tuscany region, the second in 2002 (median = 2002.21; mean = 2001.45; 95% HPD: 1990.94–2010.51) near the Livenza river (between Veneto and Friuli-Venezia-Giulia regions), the third in 2004 (median = 2004.38; mean = 2003.92; 95% HPD: 1999.61–2007.31) in Emilia-Romagna region, and the last one around 2021 (median = 2020.91; mean = 2020.84; 95% HPD: 2020.08–2021.46) in the northeast of the country (Veneto region). Following these first introductions (in particular the third one; see Figure 3), the virus then spread to Emilia-Romagna (median = 2007.79; mean = 2007.72; 95% HPD: 2006.99–2008.32), Sardinia (median = 2011.26; mean = 2011.22; 95% HPD: 2010.76–2011.57), and later on to Campania (median = 2020.49; mean = 2020.3; 95% HPD: 2017.27–2020.75) regions and to Spain (median = 2020.18; mean = 2020.06; 95% HPD: 2017.25–2020.75) (Figure 3).

Our phylogeographic analysis also indicates a clear genetic flow connecting Morocco, Spain, France, and Italy (Figure 3). This scenario was tested further by running a sensitivity analysis on three subset of the datasets, obtaining similar results (Additional file 6: Figure S1).

### Amino acid sequence analysis

**Figure 4.**
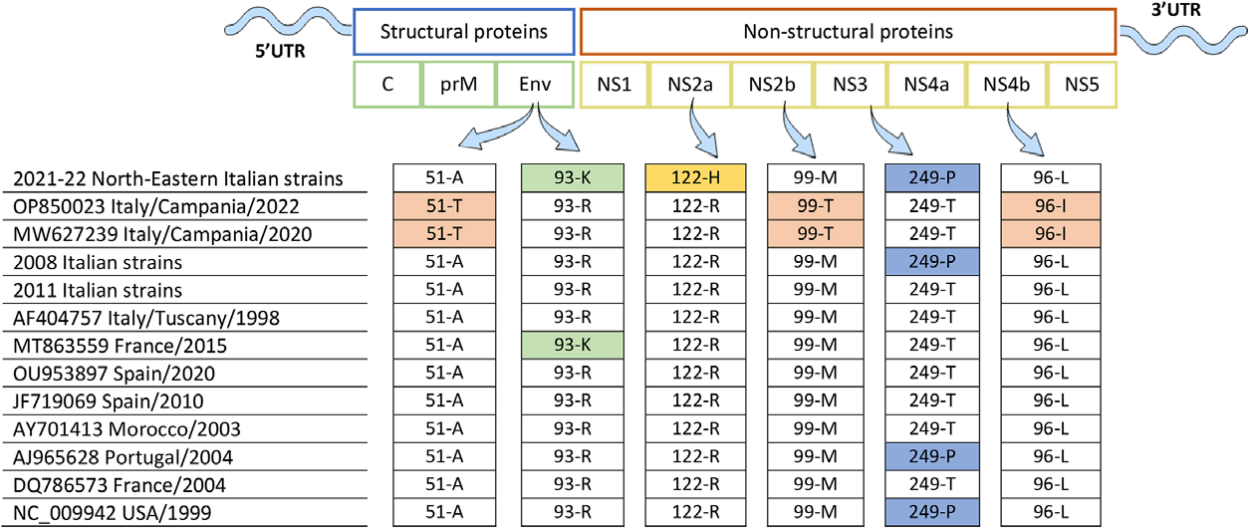
Amino acid sequence analysis. Amino acid sequence comparison between West Nile virus lineage 1 Italian strains and other Western-Mediterranean strains included in Cluster 2, other than the 1999 NC_009942 strain representative for the USA genome sequence group included in Cluster 4, is displayed. C, Capsid protein; prM, pre-membrane protein; M, Membrane protein; NS, non-structural proteins. Common point mutations (51-T, 99-T, 96-I) detected in the two Campanian strains OP850023/2022 and MW627239/2022 are displayed in pink; the point mutation 93-K of the Envelope protein detected in the 2021–2022 Northern-Eastern Italian strains and in the 2015 French strain MT863559 is displayed in light green; the 122H amino acid substitution of the NS2a protein detected in the 2021–2022 Northern-Eastern Italian strains is displayed in yellow; the 249-P point mutation is displayed in light blue.

Pairwise alignment shows the presence of a new conservative amino acid substitution (R122H) in the NS2A region of all 2021–2022 Italian WNV L1 genomes. This mutation is not present on the strain OP850023 isolated in 2022 in the Campania region (Figure 4).

Three conservative amino acid changes are observed in the E (A51T), NS2B (M99T), and NS4B (L96I) proteins of the OP850023/Campania/2022 and MW627239/Campania/2020 strains, but not in the other strains. The NS3T249P point mutation is also found in the recent 2021–2022 Italian genomes, in the group of 2008 Italian sequences, in the 2004 strain from Portugal, and in the 1999 American sequence. More details about the amino acid sequence comparison are shown in Additional file 5: Table S5.

## Discussion

WNV is the most widespread mosquito-borne pathogen in the world [44]. Currently, it is seriously affecting many European countries [45]. In Italy, WNV L2 has been the most prevalent circulating lineage in the last decade. WNV L1 strains instead have been circulating sporadically, with only few evidence reported in North-Eastern regions (2012–2014, 2017), Sardinia (2015–2016), and Campania (2020) [14] (IZS-Teramo Annual Epidemiological Bulletins, https://westnile.izs.it/j6_wnd/wndItalia, https://westnile.izs.it/j6_wnd/wndItaliaPeriodici). This was the reason why health authorities and scientists involved in the surveillance and control were greatly surprised when it reappeared in the Italian territory in the 2021–2022 epidemic seasons, even more as its emergence coincided with an increased incidence of severe WNND in humans [20, 46]. This study investigated the possible origin and evolution of the WNV L1 strains circulating in Italy, which was severely impacted by the virus compared to other EU-neighbouring countries (https://www.ecdc.europa.eu/en/west-nile-fever/surveillance-and-disease-data/historical), using two complementary approaches (phylogenetics and phylogeography) and providing insights into the viral circulation dynamics of WNV L1 in the country since 1998.

The Bayesian and maximum likelihood trees reflect the results obtained by other authors [47], who also observed the presence of seven well-supported clusters (Figure 2). Among the seven identified clusters, cluster 2 contains most of the European strains included in this study. Inside this cluster, it is worth noting the close relationship between (i) the 1998 Italian strain and the 2007–2008 group of Spanish sequences (bootstrap support = 100) (clade 3), (ii) the 2008–2009 and 2011 Italian and the 2020 Southern Spain strains (clade 1) (Figure 3), and (iii) the two Italian strains isolated in 2020 (GenBank MW627239) and 2022 (GenBank OP850023) in the Campania region to the 2008–2011 group of Italian sequences, which are also (weakly) related to a sequence from Spain (Genbank JF719069) (clade 1) [14, 48]. While, on one hand, this situation implies the presence of a corridor connecting Italy and Spain, on the other hand, taking into account the frequent silent periods observed between outbreaks, it suggests that WNV may circulate quietly for extended durations within the Western Mediterranean region [49–51].

This latter assumption is also supported by the presence of the 2008–2009 Italian group of strains, which re-emerged in Italy after 10 years of silence [51, 52]. Unlike the 1998, the 2008 incursion involved a larger area (3 regions and 8 provinces) and caused disease not only in horses but also in humans. Birds and mosquitoes were found infected too [15, 16, 51]. The virus reappeared in the same areas in 2009 and later spread to Sardinia in 2011 [17, 18]. Considering the possibility of unnoticed circulation of WNV in the area, it is not easy to figure out whether this group of Italian sequences derives from a new re-introductory event or is just the re-emergence of a strain already circulating in the past. Previous studies seem to lean towards the latter hypothesis [39]. Nevertheless, our phylogenetic tree shows a close genetic relationship between the 2004 French strains (DQ786573.1, DQ786572.1) and all Italian strains belonging to clade 1, suggesting a possible new re-introductory event from France to Italy (Figure 3). Such geographical connection is further supported by the close relatedness of the 2021–2022 North-Eastern Italian strains and the WNV L1 sequence MT863559, a strain isolated from the Natural Reserve of Camargue, in Southern France in 2015 [10], suggesting a new re-introduction to Northern Italy from Southern France. The existence of a corridor between France and Italy is also suggested by the two 2004 French strains DQ786573 and DQ786573, closely related and sister group (even if this association is not strongly supported in both maximum likelihood and clock analyses, see Figures 2 and 3) of the two major Italian clades 1 and 2 described in our phylogenetic tree.

Information on the shared ancestry assumed using phylogenetic methods has been obtained when using the phylogeographic reconstructions of WNV L1 diffusion, which provided valuable insight on the WNV L1 dynamics in a geographic and temporal context. According to our analysis, the first European WNV clade may have arisen around 1989 (median = 1989.34; mean = 1987.97; HPD 95%: 1977.15–1995.48) in a location around the Gulf of Lion, near the boundaries between France and Spain (Figure 3). All strains included in this analysis seem to be connected with this area, then spreading in two opposite directions: (i) to the Camargue region of France around 1993, moving then south-westwards (Morocco, South of Spain, and Portugal) and eastwards (Central and Northern Italy, with four independent introductions, and Southern and Central Spain) and (ii) possibly to Morocco around 1996 (see Results and Figure 3). These results must be interpreted with care and describe the entry of the clade defined by the strains included in our analysis, which is not necessarily coincident with the first entry of the virus in Europe. Having only a few samples from some European and African countries limits the power of the analysis. This is particularly evident in some situations, such as a genome of Israel (HM152775.1|Israel|31 August 2000) which is genetically associated with strains located in the Western part of the Mediterranean basin. Even though we cannot exclude a possible real close link between strains located in these two areas, the most likely scenario is an indirect connection and possible common origin of strains from sub-Saharan Africa, as suggested by a recent analysis that was oriented on clarifying the circulation of WNV between Africa and Europe [53]. Therefore, the reconstruction presented in our analysis doesn’t have to be interpreted as a successive story of linked events, as it is inherently probabilistic and dependent on the quality and quantity of the available information. It reflects the overall direction of the spread but not necessarily the precise events that happened in the past. This is particularly true for the first phases of WNV-L1 evolution that we described, where the uncertainty is much higher than for more recent events.

Despite this uncertainty, which is strongly dependent on the lack of samples from Europe and Africa, our analysis robustly supports the existence of a constant circulation among Western Mediterranean European countries, with a genetic flow between Europe and Africa, and movements from the former to the latter continent, as recently shown elsewhere [53], and also in the opposite direction. This general finding is also robust to perturbations in the dataset, as highlighted by the results of our sensitivity analysis (Additional file 6: Figure S1).

In the European continent, the great abundance of susceptible hosts and vectors that belong to the endemic fauna of the Western Mediterranean area might have indeed facilitated the rapid spread of WNV to the continent despite the diversity of habitat and climate characterizing the area [54, 55]. While the first introduction of WNV L1 in Europe can be attributed to long distance migratory birds along their Western African migration routes through Northern-western African countries, such as Senegal and Morocco [47, 53, 56, 57], the virus likely established and spread within the Western Mediterranean countries through short distance migratory birds in their shift between breeding grounds and overwintering quarters [11, 14, 49, 50, 58]. In fact, it is well known that migratory birds play a significant role in the long-range spread of WNV, getting infected during their long-distance movements across continents, and spreading the virus through different stopovers at various locations to new geographic areas that were previously unaffected by the virus [11]. Once infected, some endemic bird species, more susceptible than others, might act as amplifying hosts, developing high levels of viraemia able to further infect mosquitoes, and increasing the likelihood of transmission to other animals or humans [59].

As shown by Pesko and Ebel [60], changes to the WNV genome could also affect the transmission routes, host immune responses, and virus pathogenesis [61]. In many epidemics, including those occurring in Italy, there were some WNV L1 strains capable of causing severe neurological cases in humans, horses, and birds [62] (https://westnile.izs.it/j6_wnd/home). Our pairwise alignment evidenced several point mutations, which can be screened in future studies to test their potential in increasing virulence (Figure 4 and Additional file 5: Table S5) [20]. The ecological and climatic Italian conditions of the 2021–2022 seasons might have created a favourable environment for a strong viral circulation and many replication events and, in turn, the appearance of new genetic variants among the group of Italian strains [20]. These studies might help to understand the mechanisms underlying clinical signs of WNV in humans, giving a possible explanation for the new WNV L1 North-Eastern Italian region’s pathogenic phenotype. Moreover, they may help the comprehension of the mechanisms at the basis of viral evolution, viral fitness, and host adaptation. Understanding how these factors shape WNV L1 diverse viral populations is crucial to uncover the virus evolution, epidemiology, and prevalence.

Interestingly, the genome sequence OP850023 obtained from a horse in the Campania region in October 2022 is not included in the group of North-Eastern Italian sequences, although coming from the same epidemic season. It is closely related (posterior probability = 1, Figure 3) to the sequence MW627239, obtained also in the Campania region from a Northern goshawk 2 years before (October 2020) [14]. They do not carry the NS2AR122H point mutation reported in all 2021–2022 North-Eastern strains but show three common new amino acid substitutions (EA51T, NS2BM99T, and NS4BL96I) (Figure 4 and Additional file 5: Table S5). It is not simple to assess the exact source of the introduction of the virus in the Campania region. Even though our maximum likelihood tree placed the Campanian strains close to the 2008–2011 group of Italian sequences and to a 2010 Spanish sequence (Genbank JF719069) (clade 1), suggesting an introduction to the Campania region from Spain or Italy (Northern Italy/2008, Sardinia/2011), the latter also supported by our phylogeographic analysis (Figure 3). Once introduced in Campania, these strains might have found a new ecological niche, establishing an intra-endemic circulation [14]. Another possibility is that the mutations observed may have accumulated before the invasion and could therefore be the result of other ecological processes and/or a founder effect.

## Conclusion

In this study, the 31 new Italian genome sequences obtained by the IZS-Teramo from 2008 to 2022 were used to uncover the phylogenetic and phylogeographic relationships of WNV L1 Italian strains with other strains circulating worldwide. We clarified the spatial and temporal dynamics of WNV L1 in Italy in the last 24 years and identified the presence of two diverse strains currently circulating in the country (one in the North-East and one circulating intra-regionally in the Campania region). Furthermore, we found that WNV L1 spreads through an ideal line that connects Morocco, Spain, Southern France, and Northern Italy, with further diffusion between diverse regions of the Italian territory and possible events of endemization. These results help clarify the WNV L1 dynamics in Europe, provide a new consistent dataset that can be used as reference data for future WNV investigations, and highlight the strong need of constant and coordinated surveillance activities among European and African countries.

Due to gaps in genomic surveillance data for WNV in Europe and a limited number of newly generated genomes, our results stressed the importance to further intensify WNV surveillance in the Mediterranean countries and, in particular, in areas where the virus might find favourable conditions for its endemic circulation, either in wetlands inhabited by birds and mosquitoes (Southern France and Southern and Eastern Spain) [10, 48] or in areas where the circulation might seem reduced or absent. This will contribute to building a more consistent and homogeneous European dataset that could tackle the uncertainties related to the WNV dynamics.

## Supporting information

Silverj et al. supplementary material 1 Silverj et al. supplementary material

Silverj et al. supplementary material 2 Silverj et al. supplementary material

Silverj et al. supplementary material 3 Silverj et al. supplementary material

Silverj et al. supplementary material 4 Silverj et al. supplementary material

Silverj et al. supplementary material 5 Silverj et al. supplementary material

Silverj et al. supplementary material 6 Silverj et al. supplementary material

Silverj et al. supplementary material 7 Silverj et al. supplementary material

## Data Availability

All scripts used to perform the analyses are available at the GitHub repository: https://github.com/andrea-silverj/WNV-L1_IT. All data that support the findings of the study are available from the material associated with Figshare data repository (Project n. 157871). Particularly, meta-data used for phylogenetic and phylogeographic analyses and Run details reported for each of the 31 IZS-samples and negative controls, and amino acid sequence comparison can be found at https://doi.org/10.6084/m9.figshare.21947681.v4; Tree files and sequence alignment used for phylogenetic analyses can be found at https://doi.org/10.6084/m9.figshare.21940898.v3; Phylogeographic inference tree files, geographic coordinates, and videos can be found at (i) https://doi.org/10.6084/m9.figshare.21940931.v2 (sub-selected dataset) and (ii) https://doi.org/10.6084/m9.figshare.23631735.v2 (complete dataset). This material is published under the Creative Commons Attribution 4.0 International (CC BY 4.0).

## Supplementary material

The supplementary material for this article can be found at http://doi.org/10.1017/S0950268824001420.
