# Supplementary figures and images for: Origin and evolution of West Nile virus lineage 1 in Italy
Source: Epidemiol Infect. 2024 Dec 2;152:e150. doi: 10.1017/S0950268824001420 (PMC11626449; doi:10.1017/S0950268824001420)

**A**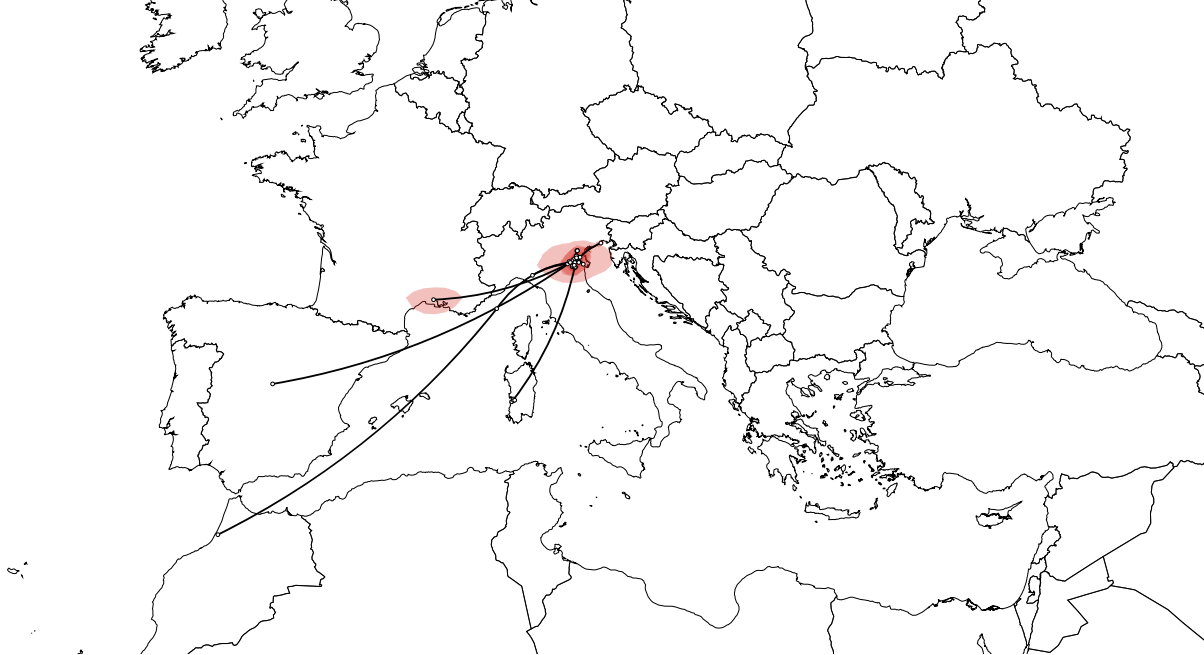**B**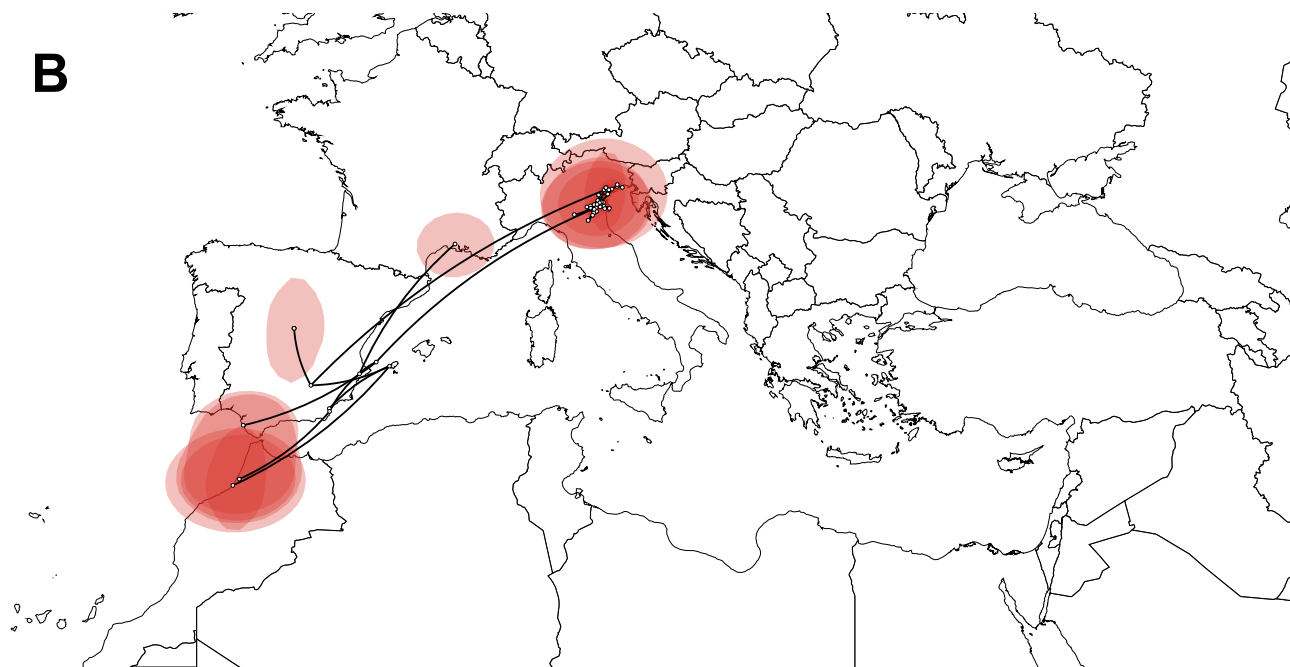**C**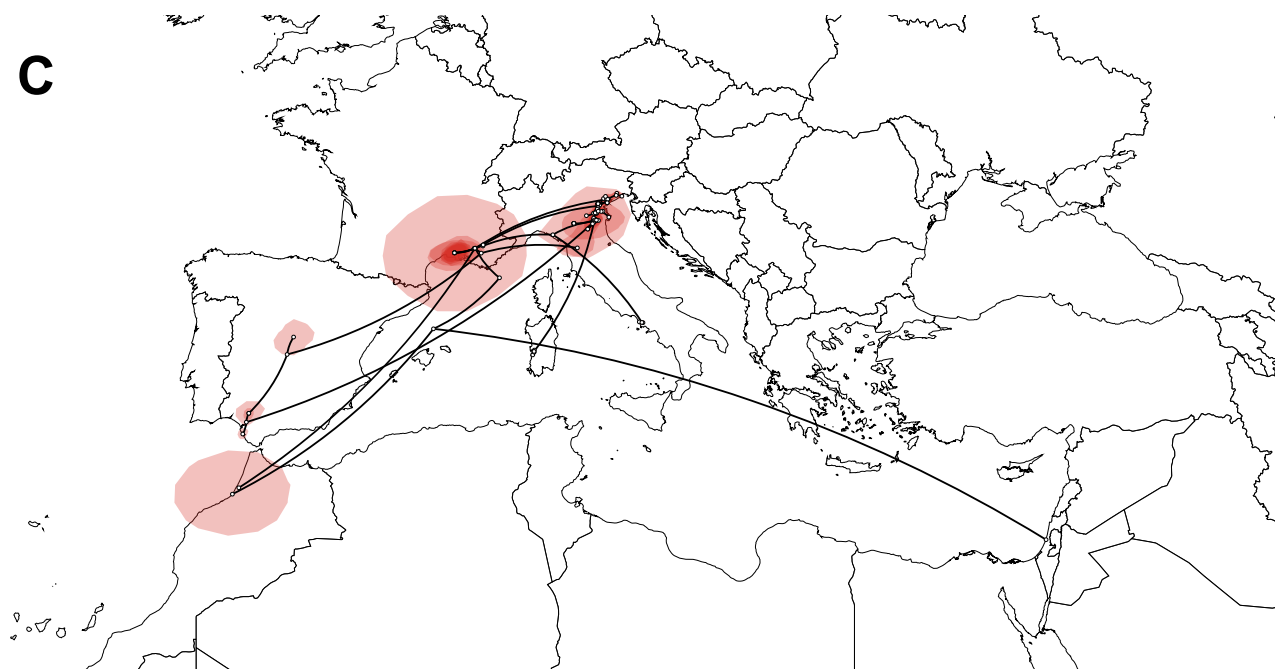

Supplement: Silverj et al. supplementary material 1 — Silverj et al. supplementary material [file S0950268824001420sup001.pdf]
