# Supplementary material for: Origin and evolution of West Nile virus lineage 1 in Italy
Source: Epidemiol Infect. 2024 Dec 2;152:e150. doi: 10.1017/S0950268824001420 (PMC11626449; doi:10.1017/S0950268824001420)
Supplement: Silverj et al. supplementary material 3 — Silverj et al. supplementary material [file S0950268824001420sup003.pdf]

Primer ID	Name of the primer	5'- 3' sequence
FR #1	WNIT98_5_END_F	AGTAGTTCGCCTGTGTGAGCTGACAAAC
	WNIT98_5_END_R	CGCCTTCGAGAACCAAATCCAC
FR #2	WN_IT98/128_F	GCCGGGCTGTCAATATGCTAAAAC
	WN_IT98/1456_R	CCTGAGTGGCCCCAATCTGTGT
FR #3	WN_IT98/1331_F	CCACCAAGGCAACAGGAA
	WN_IT98/2458_R	TCACGGAGAGGAAGAGCAGGAC
FR #4	WN_IT98/2017_F	CCGGTAGGCAGATTGGTCA
	WN_IT98/3270_R	TGGGCCCTGGTTTTGTGTCTTGTA
FR #5	WN_IT98/3022_F	GGAACGGCCGTCAAAAACAAC
	WN_IT98/4461_R	ATCATCAAGCCGCACATCAACTCT
FR #6	WN_IT98/4429_F	TCGAGCGAAAGAGTTGATGTGC
	WN_IT98/5514_R	AATATAACCTCTTGCTGCGATGCT
FR #7	WN_IT98/5489_F	CTAGCATCGCAGCAAGAGGTTAT
	WN_IT98/6621_R	GAGAGCATCCGGCAGTTCC
FR #8	WN_IT98/6465_F	GCGTTCTCAGATAGGGCTCATTG
	WN_IT98/7493_R	TTCACAGACGGGTTCACTACTACT
FR #9	WN_IT98/7345_F	GGAATCATGAAAAACGCTGTAGTG
	WN_IT98/8513_R	TTGCTGGTGTCTGAGTTGAGTAGG
FR #10	WN_IT98/8483_F	GGAAACCCCTACTCAACTCAGACAC
	WN_IT98/9572_R	ACTCCTTCCCCTTCCATCATCCT
FR #11	WN_IT98/8426_F	GGAAGGGACCCCAATACGAG
	WN_IT98/9714_R	GCGATCATCCAGAGGCTTTACC
FR #12	WN_IT98/9683_F	ATCACTGCGTGGTAAAGCCTCTGG
	WN_IT98/10809_R	CCTCTAGTCCTTTCGCCCTGGTTA
FR #13	WN_IT98_3_END_F	CACTTTCTCAACGCCATGTCAA
	WN_IT98_3_END_R	AGATCCTGTGTTCTCGCACC
