# Supplementary material for: Origin and evolution of West Nile virus lineage 1 in Italy
Source: Epidemiol Infect. 2024 Dec 2;152:e150. doi: 10.1017/S0950268824001420 (PMC11626449; doi:10.1017/S0950268824001420)
Supplement: Silverj et al. supplementary material 4 — Silverj et al. supplementary material [file S0950268824001420sup004.pdf]

Accession number	Host	Host category	Isolation Source	Continent	Country	Region	Collection Date	TreeID
AF404757.1	Equus caballus	Horse	NA	Europe	Italy	Tuscany	3/09/1998	AF404757.1|Italy|1998/09/03
MW627239*	Accipiter gentilis	Bird	NA	Europe	Italy	Campania	1/10/2020	MW627239.1|Italy|2020/10/01
MW915462.1	Accipiter gentilis	Bird	Brain	Europe	Spain	NA	15/9/2017	MW915462.1|Spain|2017/09/15
MZ605381.2	Phoenicopterus roseus	Bird	NA	North America	USA	NA	15/6/1999	MZ605381.2|USA|1999/06/15
MT905060.1	Equus caballus	Horse	NA	South America	Brazil	NA	13/6/2019	MT905060.1|Brazil|2019/06/13
MT863559.1	Equus caballus	Horse	NA	Europe	France	Provence-Alpes-Côte d'Azur	3/10/2015	MT863559.1|France|2015/10/03
MT967998.1	Culicidae	Mosquito	NA	North America	USA	NA	15/6/2014	MT967998.1|USA|2014/06/15
MT968022.1	Culicidae	Mosquito	NA	North America	USA	NA	15/6/2018	MT968022.1|USA|2018/06/15
LC489409.1	Crocodylus niloticus	Reptile	NA	Africa	Zambia	NA	15/6/2019	LC489409.1|Zambia|2019/06/15
MN149538.1	Acrocephalus dumetorum	Bird	NA	Russia	Russia	NA	15/5/2006	MN149538.1|Russia|2006/05/15
MH819446.1	Culex	Mosquito	Pooled insects	North America	Canada	NA	6/10/2015	MH819446.1|Canada|2015/10/06
MH819448.1	Culex	Mosquito	Pooled insects	North America	Canada	NA	31/8/2005	MH819448.1|Canada|2005/08/31
MH819449.1	Culex	Mosquito	Pooled insects	North America	Canada	NA	22/9/2014	MH819449.1|Canada|2014/09/22
MH819450.1	Culex	Mosquito	Pooled insects	North America	Canada	NA	16/8/2016	MH819450.1|Canada|2016/08/16
MH819451.1	Culex	Mosquito	Pooled insects	North America	Canada	NA	12/8/2005	MH819451.1|Canada|2005/08/12

MH819452.1	Culex	Mosquito	Pooled insects	North America	Canada	NA	15/9/2015	MH819452.1|Canada|2015/09/15
MH819453.1	Culex	Mosquito	Pooled insects	North America	Canada	NA	9/8/2016	MH819453.1|Canada|2016/08/09
MH819454.1	Culex	Mosquito	Pooled insects	North America	Canada	NA	29/8/2006	MH819454.1|Canada|2006/08/29
MH819455.1	Culex	Mosquito	Pooled insects	North America	Canada	NA	30/8/2006	MH819455.1|Canada|2006/08/30
MH819456.1	Culex	Mosquito	Pooled insects	North America	Canada	NA	9/9/2013	MH819456.1|Canada|2013/09/09
MH819457.1	Culex	Mosquito	Pooled insects	North America	Canada	NA	19/8/2005	MH819457.1|Canada|2005/08/19
MH819458.1	Culex	Mosquito	Pooled insects	North America	Canada	NA	25/8/2005	MH819458.1|Canada|2005/08/25
MH819459.1	Culex	Mosquito	Pooled insects	North America	Canada	NA	4/9/2014	MH819459.1|Canada|2014/09/04
MH819460.1	Culex	Mosquito	Pooled insects	North America	Canada	NA	19/8/2013	MH819460.1|Canada|2013/08/19
MH819461.1	Culex	Mosquito	Pooled insects	North America	Canada	NA	7/9/2004	MH819461.1|Canada|2004/09/07
MH819462.1	Culex	Mosquito	Pooled insects	North America	Canada	NA	9/9/2015	MH819462.1|Canada|2015/09/09
MH819463.1	Culex	Mosquito	Pooled insects	North America	Canada	NA	21/8/2014	MH819463.1|Canada|2014/08/21
MH819464.1	Culex	Mosquito	Pooled insects	North America	Canada	NA	15/9/2004	MH819464.1|Canada|2004/09/15
MH819465.1	Culex	Mosquito	Pooled insects	North America	Canada	NA	9/9/2015	MH819465.1|Canada|2015/09/09
MH819466.1	Culex	Mosquito	Pooled insects	North America	Canada	NA	16/8/2016	MH819466.1|Canada|2016/08/16
MH819467.1	Culex	Mosquito	Pooled insects	North America	Canada	NA	19/8/2013	MH819467.1|Canada|2013/08/19
MH819468.1	Culex	Mosquito	Pooled insects	North America	Canada	NA	8/8/2004	MH819468.1|Canada|2004/08/08

MH819469.1	Aedes vexans	Mosquito	Pooled insects	North America	Canada	NA	29/8/2006	MH819469.1|Canada|2006/08/29
MH819470.1	Culex	Mosquito	Pooled insects	North America	Canada	NA	15/9/2014	MH819470.1|Canada|2014/09/15
MH819471.1	Culex	Mosquito	Pooled insects	North America	Canada	NA	7/9/2004	MH819471.1|Canada|2004/09/07
MH507645.1	Buteo lineatus	Bird	Tissue	North America	USA	NA	8/4/2009	MH507645.1|USA|2009/04/08
MH507712.1	Aphelocoma californica	Bird	Tissue	North America	USA	NA	18/8/2008	MH507712.1|USA|2008/08/18
MH507747.1	Corvus brachyrhynchos	Bird	Tissue	North America	USA	NA	3/7/2017	MH507747.1|USA|2017/07/03
MH507748.1	Corvus brachyrhynchos	Bird	Tissue	North America	USA	NA	11/7/2017	MH507748.1|USA|2017/07/11
MH507749.1	Corvus brachyrhynchos	Bird	Tissue	North America	USA	NA	13/7/2017	MH507749.1|USA|2017/07/13
MH507822.1	Culex tarsalis	Mosquito	Pooled insects	North America	USA	NA	29/6/2017	MH507822.1|USA|2017/06/29
MH507838.1	Aphelocoma californica	Bird	Tissue	North America	USA	NA	7/6/2007	MH507838.1|USA|2007/06/07
MH507846.1	Aphelocoma californica	Bird	Tissue	North America	USA	NA	29/6/2007	MH507846.1|USA|2007/06/29
MH507850.1	Aphelocoma californica	Bird	Tissue	North America	USA	NA	9/7/2007	MH507850.1|USA|2007/07/09
MH507889.1	Corvus brachyrhynchos	Bird	Tissue	North America	USA	NA	4/7/2017	MH507889.1|USA|2017/07/04
MH508025.1	Culex tarsalis	Mosquito	Pooled insects	North America	USA	NA	4/8/2011	MH508025.1|USA|2011/08/04
MH508037.1	Culex quinquefasciatus	Mosquito	Pooled insects	North America	USA	NA	28/6/2017	MH508037.1|USA|2017/06/28
AF196835.2	Aves	Bird	Tissue	America	USA	NA	31/08/1999	AF196835.2|USA|1999/08/31
AF260968.1	Homo sapiens	Human	Blood	Africa	Egypt	NA	31/08/1951	AF260968.1|Egypt|31/08/1951
AF260969.1	Aves	Bird	NA	Europe	Romania	NA	05/10/1996	AF260969.1|Romania|05/10/1996

<table>
<tr><td>AF317203.1</td><td>Homo sapiens</td><td>Human</td><td>Brain</td><td>Russia</td><td>Russia</td><td>NA</td><td>15/09/1999</td><td>AF317203.1|Russia|15/09/1999</td></tr>
<tr><td>AF481864.1</td><td>Aves</td><td>Bird</td><td>NA</td><td>West Asia</td><td>Israel</td><td>NA</td><td>28/08/1998</td><td>AF481864.1|Israel|1998/08/28</td></tr>
<tr><td>AM404308.1</td><td>Culex</td><td>Mosquito</td><td>Pooled insects</td><td>Europe</td><td>Portugal</td><td>NA</td><td>31/08/197</td><td>AM404308.1|Portugal|31/08/1971</td></tr>
<tr><td>AY262283.1</td><td>Culex</td><td>Mosquito</td><td>Pooled insects</td><td>Africa</td><td>Kenya</td><td>NA</td><td>31/08/1998</td><td>AY262283.1|Kenya|31/08/1998</td></tr>
<tr><td>AY268133.1</td><td>Homo sapiens</td><td>Human</td><td>Brain</td><td>Africa</td><td>Tunisia</td><td>NA</td><td>31/08/1997</td><td>AY268133.1|Tunisia|31/08/1997</td></tr>
<tr><td>AY277252.1</td><td>Homo sapiens</td><td>Human</td><td>Brain</td><td>Russia</td><td>Russia</td><td>NA</td><td>31/08/1998</td><td>AY277252.1|Russia|31/08/1999</td></tr>
<tr><td>AY278442.1</td><td>Homo sapiens</td><td>Human</td><td>Blood</td><td>Russia</td><td>Russia</td><td>NA</td><td>31/08/2000</td><td>AY278442.1|Russia|31/08/2000</td></tr>
<tr><td>AY289214.1</td><td>Homo sapiens</td><td>Human</td><td>NA</td><td>America</td><td>USA</td><td>NA</td><td>31/08/2002</td><td>AY289214.1|USA|31/08/2002</td></tr>
<tr><td>DQ118127.1</td><td>Aves</td><td>Birds</td><td>NA</td><td>Europe</td><td>Hungary</td><td>NA</td><td>15/08/2003</td><td>DQ118127.1|Hungary|15/08/2003</td></tr>
<tr><td>EU249803.1</td><td>Chiroptera</td><td>Bat</td><td>NA</td><td>Asia</td><td>India</td><td>NA</td><td>31/08/1968</td><td>EU249803.1|India|31/08/1968</td></tr>
<tr><td>GU827998.1</td><td>Blue jay</td><td>Bird</td><td>NA</td><td>America</td><td>USA</td><td>Texas</td><td>31/08/2002</td><td>GU827998.1|USA|31/08/2002</td></tr>
<tr><td>KU573077.1</td><td>Pica pica</td><td>Bird</td><td>NA</td><td>Europe</td><td>Italy</td><td>Emilia-Romagna</td><td>15/6/2008</td><td>KU573077.1|Italy|2008/06/15</td></tr>
<tr><td>KU573078.1</td><td>Culex pipiens</td><td>Mosquito</td><td>NA</td><td>Europe</td><td>Italy</td><td>Emilia-Romagna</td><td>15/6/2009</td><td>KU573078.1|Italy|2009/06/15</td></tr>
<tr><td>KU573079.1</td><td>Pica pica</td><td>Bird</td><td>NA</td><td>Europe</td><td>Italy</td><td>Emilia-Romagna</td><td>15/6/2009</td><td>KU573079.1|Italy|2009/06/15</td></tr>
<tr><td>MF175839.1</td><td>Culex quinquefasciatus</td><td>Mosquito</td><td>NA</td><td>North America</td><td>USA</td><td>NA</td><td>28/8/2014</td><td>MF175839.1|USA|2014/08/28</td></tr>
<tr><td>MF175842.1</td><td>Culex quinquefasciatus</td><td>Mosquito</td><td>NA</td><td>North America</td><td>USA</td><td>NA</td><td>17/10/2014</td><td>MF175842.1|USA|2014/10/17</td></tr>
<tr><td>MH170239.1</td><td>Culex tarsalis</td><td>Mosquito</td><td>NA</td><td>North America</td><td>USA</td><td>NA</td><td>15/6/2006</td><td>MH170239.1|USA|2006/06/15</td></tr>
</table>

MH170263.1	Cyanocitta cristata	Bird	NA	North America	USA	NA	15/6/2001	MH170263.1|USA|2001/06/15
MH170265.1	Culex quinquefasciatus	Mosquito	NA	North America	USA	NA	15/6/2004	MH170265.1|USA|2004/06/15
MG004540.1	Culex quinquefasciatus	Mosquito	Pooled insects	North America	USA	NA	12/5/2015	MG004540.1|USA|2015/05/12
KY703854.1	Culex poicilipes	Mosquito	NA	Africa	Senegal	NA	15/6/1990	KY703854.1|Senegal|1990/06/15
MF797870.1	Homo sapiens	Human	Urine	Europe	Cyprus	NA	15/7/2016	MF797870.1|Cyprus|2016/07/15
KY229069.1	Culex pipiens	Mosquito	NA	North America	USA	NA	7/8/2012	KY229069.1|USA|2012/08/07
KY229073.1	Culex pipiens	Mosquito	NA	North America	USA	NA	21/8/2012	KY229073.1|USA|2012/08/21
KU978766.1	Phoenicopterus ruber	Bird	NA	South America	Colombia	NA	15/7/2008	KU978766.1|Colombia|2008/07/15
KU978768.1	Hyalomma	Tick	NA	Russia	Russia	NA	15/6/1963	KU978768.1|Russia|1963/06/15
KU978769.1	Corvus	Bird	NA	North America	Mexico	NA	30/5/2003	KU978769.1|Mexico|2003/05/30
KX547277.1	Culex	Mosquito	NA	North America	USA	NA	23/9/2004	KX547277.1|USA|2004/09/23
KX547394.1	Coquillettidia	Mosquito	NA	North America	USA	NA	1/8/2006	KX547394.1|USA|2006/08/01
KX547412.1	Aedes sp.	Mosquito	NA	North America	USA	NA	30/8/2011	KX547412.1|USA|2011/08/30
KX547538.1	Culex	Mosquito	NA	North America	USA	NA	15/8/2001	KX547538.1|USA|2001/08/15
KM012173.1	Homo sapiens	Human	NA	North America	USA	NA	15/6/2012	KM012173.1|USA|2012/06/15
KU588135.1	Camelus dromedarius	Camel	NA	West Asia	United Arab Emirates	NA	15/6/2015	KU588135.1|United Arab Emirates|2015/06/15
KT163243.1	NA	NA	NA	India	India	NA	1/1/2015	KT163243.1|India|2015/01/01
KR107956.1	NA	NA	NA	Europe	Belgium	NA	15/6/2014	KR107956.1|Belgium|2014/06/15

KJ958922.1	Equus caballus	Horse	Blood	Europe	Turkey	NA	15/6/2011	KJ958922.1|Turkey|2011/06/15
KF823807.1	Homo sapiens	Human	Urine	Europe	Italy	Veneto	15/9/2013	KF823807.1|Italy|2013/09/15
JX442279.1	Culex pipiens pipiens	Mosquito	NA	China	China	NA	15/6/2011	JX442279.1|China|2011/06/15
KJ501360.1	Cyanocitta cristata	Bird	Feather	North America	USA	NA	15/6/2003	KJ501360.1|USA|2003/06/15
KJ501394.1	Corvus brachyrhynchos	Bird	Kidney, Spleen, Abdominal cavity	North America	USA	NA	15/6/2001	KJ501394.1|USA|2001/06/15
KJ501430.1	Pelecanus erythrorhynchos	Bird	Feather	North America	USA	NA	15/6/2006	KJ501430.1|USA|2006/06/15
KJ501443.1	Corvus brachyrhynchos	Bird	Kidney, Spleen, Abdominal cavity	North America	USA	NA	15/6/2001	KJ501443.1|USA|2001/06/15
KJ501516.1	Corvus brachyrhynchos	Bird	Kidney, Spleen, Abdominal cavity	North America	USA	NA	15/6/2001	KJ501516.1|USA|2001/06/15
KF367469.1	Phoenicopterus roseus	Bird	Heart	North America	British Virgin Islands	NA	30/3/2013	KF367469.1|British Virgin Islands|2013/03/30
KF647253.1	Homo sapiens	Human	Blood	Europe	Italy	Veneto	15/8/2013	KF647253.1|Italy|2013/08/15
KF234080.1	Homo sapiens	Human	NA	Europe	Italy	Emilia-Romagna	15/6/2009	KF234080.1|Italy|2009/06/15
KC954092.1	Homo sapiens	Human	Urine	Europe	Italy	Veneto	15/9/2012	KC954092.1|Italy|2012/09/15
KC407667.1	Mus musculus	Mouse	NA	Europe	Spain	NA	15/6/2007	KC407667.1|Spain|2007/06/15
KC601756.1	Homo sapiens	Human	NA	India	India	NA	15/2/2011	KC601756.1|India|2011/02/15

KC243146.1	Rhipicephalus pulchellus	Tick	NA	Africa	Kenya	NA	27/11/2010	KC243146.1|Kenya|2010/11/27
JX015518.1	Culex quinquefasciatus	Mosquito	NA	North America	Mexico	NA	9/9/2008	JX015518.1|Mexico|2008/09/09
JX015520.1	Culex quinquefasciatus	Mosquito	NA	North America	Mexico	NA	11/8/2009	JX015520.1|Mexico|2009/08/11
JX556213.1	Homo sapiens	Human	NA	Europe	Italy	Veneto	3/8/2012	JX556213.1|Italy|2012/08/03
JQ928174.1	Homo sapiens	Human	Blood	Europe	Italy	Veneto	15/9/2011	JQ928174.1|Italy|2011/09/15
JQ928175.1	Homo sapiens	Human	Urine	Europe	Italy	Veneto	15/9/2011	JQ928175.1|Italy|2011/09/15
JX041628.1	Ornithodoros capensis	Tick	NA	West Asia	Azerbaijan	NA	15/6/1970	JX041628.1|Azerbaijan|1970/06/15
JX041629.1	Aves	Bird	NA	West Asia	Azerbaijan	NA	15/6/1967	JX041629.1|Azerbaijan|1967/06/15
JX041630.1	Aves	Bird	NA	West Asia	Azerbaijan	NA	15/6/1967	JX041630.1|Azerbaijan|1967/06/15
JX041633.1	Hyalomma plumbeum plumbeum	Tick	NA	Russia	Russia	NA	15/6/1963	JX041633.1|Russia|1963/06/15
JX041634.1	Homo sapiens	Human	NA	Russia	Russia	NA	15/6/1999	JX041634.1|Russia|1999/06/15
JN716371.1	Phoenicopterus ruber	Bird	NA	South America	Colombia	NA	15/7/2008	JN716371.1|Colombia|2008/07/15
JN858069.1	Homo sapiens	Human	Urine	Europe	Italy	Marche	15/9/2011	JN858069.1|Italy|2011/09/15
JF707789.1	Culex perexiguus	Mosquito	NA	Europe	Spain	Andalucia	15/6/2008	JF707789.1|Spain|2008/06/15
JN051152.1	Corvus corax	Bird	NA	North America	Mexico	NA	15/5/2003	JN051152.1|Mexico|2003/05/15
JN051153.1	Corvus corax	Bird	NA	North America	Mexico	NA	15/5/2003	JN051153.1|Mexico|2003/05/15
JF719065.1	Corvidae	Bird	NA	Europe	Italy	Emilia-Romagna	15/10/2008	JF719065.1|Italy|2008/10/15

JF719066.1	Corvidae	Bird	NA	Europe	Italy	Emilia-Romagna	15/9/2008	JF719066.1|Italy|2008/09/15
JF719067.1	Laridae	Bird	NA	Europe	Italy	Emilia-Romagna	15/11/2009	JF719067.1|Italy|2009/11/15
JF719068.1	Corvidae	Bird	NA	Europe	Italy	Emilia-Romagna	15/10/2009	JF719068.1|Italy|2009/10/15
JF719069.1	Equus caballus	Horse	Brain	Europe	Spain	Andalusia	15/8/2010	JF719069.1|Spain|2010/08/15
OU953895.1	Homo sapiens	Human	NA	Europe	Spain	Andalusia	13/08/2020	OU953895.1|Spain|2020/08/13
OU953896.1	Homo sapiens	Human	NA	Europe	Spain	Andalusia	09/09/2020	OU953896.1|Spain|2020/09/09
OU953897.1	Homo sapiens	Human	NA	Europe	Spain	Andalusia	13/08/2020	OU953897.1|Spain|2020/08/13
JF415928.1	Culex quinquefasciatus	Mosquito	NA	North America	USA	NA	15/6/2009	JF415928.1|USA|2009/06/15
JF972636.1	Culiseta melanura	Mosquito	NA	North America	USA	NA	15/6/2008	JF972636.1|USA|2008/06/15
JF488088.1	Culex pipiens	Mosquito	NA	North America	USA	NA	15/6/2004	JF488088.1|USA|2004/06/15
JF488091.1	Culex salinarius	Mosquito	NA	North America	USA	NA	15/6/2004	JF488091.1|USA|2004/06/15
HM051416.1	Homo sapiens	Human	NA	West Asia	Israel	NA	15/6/1953	HM051416.1|Israel|1953/06/15
GQ851606.1	NA	NA	NA	Africa	Senegal	NA	15/6/1979	GQ851606.1|Senegal|1979/06/15
GQ851607.1	NA	NA	NA	Africa	Nigeria	NA	15/6/1965	GQ851607.1|Nigeria|1965/06/15
GQ851608.1	NA	NA	NA	Africa	Central African Republic	NA	15/6/1967	GQ851608.1|Central African Republic|1967/06/15
HM756653.1	Culex pipiens	Mosquito	NA	North America	USA	NA	15/6/2003	HM756653.1|USA|2003/06/15
HM756660.1	Accipiter cooperii	Bird	Brain	North America	USA	NA	15/6/2008	HM756660.1|USA|2008/06/15

HM152773.1	Homo sapiens	Human	NA	West Asia	Israel	NA	15/6/2000	HM152773.1|Israel|2000/06/15
HM152775.1	Homo sapiens	Human	NA	West Asia	Israel	Tel-Aviv district	15/6/2000	HM152775.1|Israel|2000/06/15
GU011992.2	Homo sapiens	Human	NA	Europe	Italy	Veneto	15/5/2009	GU011992.2|Italy|2009/05/15
GQ379156.1	Corvus	Bird	NA	North America	USA	NA	15/7/2001	GQ379156.1|USA|2001/07/15
GQ379160.1	Equus caballus	Horse	Brain	South America	Argentina	NA	15/2/2006	GQ379160.1|Argentina|2006/02/15
GQ379161.1	Equus caballus	Horse	Brain	South America	Argentina	NA	15/2/2006	GQ379161.1|Argentina|2006/02/15
FJ766331.1	Aquila chrysaetos	Bird	NA	Europe	Spain	NA	15/10/2007	FJ766331.1|Spain|2007/10/15
FJ766332.1	Aquila chrysaetos	Bird	NA	Europe	Spain	NA	15/10/2007	FJ766332.1|Spain|2007/10/15
FJ483548.1	Corvidae	Bird	NA	Europe	Italy	Emilia-Romagna	15/6/2008	FJ483548.1|Italy|2008/06/15
FJ483549.1	Corvidae	Bird	NA	Europe	Italy	Emilia-Romagna	15/6/2008	FJ483549.1|Italy|2008/06/15
AY268132.1	Equus caballus	Horse	NA	Europe	France	Provence-Alpes-Côte d’Azur	31/08/2000	AY268132.1|France|2000/08/31
DQ786572.1	Passer domesticus	Bird	Brain cell	Europe	France	Provence-Alpes-Côte d’Azur	18/10/2004	DQ786572.1|France|2004/10/18
DQ786573.1	Pica pica	Bird	Brain cell	Europe	France	Provence-Alpes-Côte d’Azur	21/10/2004	DQ786573.1|France|2004/10/21
DQ431699.1	Homo sapiens	Human	Blood	North America	USA	NA	15/6/2003	DQ431699.1|USA|2003/06/15
AY701412.1	Equus caballus	Horse	Brain	Africa	Morocco	NA	15/6/1996	AY701412.1|Morocco|1996/06/15
AY701413.1	Equus caballus	Horse	Brain	Africa	Morocco	Rabat-Salé-Kenitra	15/6/2003	AY701413.1|Morocco|2003/06/15

AJ965628.2	-	Mosquito	NA	Europe	Portugal	Algarve	15/07/2004	AJ965628.2|Portugal|2004/07/15
OP009520	Collared dove	Bird	NA	Europe	Italy	Veneto	15/06/2021	OP009520.1|Italy|2021/06/15
OP009522	Culex pipiens	Mosquito	Pooled insects	Europe	Italy	Veneto	16/08/2021	OP009522.1|Italy|2021/08/21
OP009523	Culex pipiens	Mosquito	Pooled insects	Europe	Italy	Veneto	16/08/2021	OP009523.1|Italy|2021/08/21
OP009524	Culex pipiens	Mosquito	Pooled insects	Europe	Italy	Veneto	16/08/2021	OP009524.1|Italy|2021/08/21
OP009521	Culex pipiens	Mosquito	Pooled insects	Europe	Italy	Veneto	23/08/2021	OP009521.1|Italy|2021/08/21
OP009525	Culex pipiens	Mosquito	Pooled insects	Europe	Italy	Veneto	21/06/2021	OP009525.1|Italy|2021/08/21
OP734262*	Culex pipiens	Mosquito	Homogenate	Europe	Italy	Veneto	21/6/2022	OP734262|Italy|2022/06/21
OP734263*	Culex pipiens	Mosquito	Homogenate	Europe	Italy	Veneto	7/7/2022	OP734263|Italy|2022/07/07
OP734264*	Pica pica	Bird	Homogenate	Europe	Italy	Veneto	28/6/2022	OP734264|Italy|2022/06/28
OP734265*	Phalacrocorax carbo	Bird	Homogenate	Europe	Italy	Veneto	7/7/2022	OP734265|Italy|2022/07/07
OP734266*	Culex pipiens	Mosquito	Homogenate	Europe	Italy	Emilia-Romagna	12/7/2022	OP734266|Italy|2022/07/12
OP734267*	Culex pipiens	Mosquito	Homogenate	Europe	Italy	Emilia-Romagna	12/7/2022	OP734267|Italy|2022/07/12
OP734268*	Culex pipiens	Mosquito	Homogenate	Europe	Italy	Veneto	7/7/2022	OP734268|Italy|2022/07/07
OP734269*	Corvus cornix	Bird	Homogenate	Europe	Italy	Veneto	18/7/2022	OP734269|Italy|2022/07/18
OP734270*	Culex pipiens	Mosquito	Homogenate	Europe	Italy	Emilia-Romagna	19/7/2022	OP734270|Italy|2022/07/13
OP734271*	Corvus cornix	Bird	Homogenate	Europe	Italy	Veneto	13/7/2022	OP734271|Italy|2022/07/13
OP734272*	Passer domesticus	Bird	Homogenate	Europe	Italy	Veneto	4/7/2022	OP734272|Italy|2022/07/04

OP734274*	Culex pipiens	Mosquito	Homogenate	Europe	Italy	Veneto	14/7/2022	OP734274|Italy|2022/07/14
OP850021*	Corvus cornix	Bird	Homogenate	Europe	Italy	Lombardy	12/9/2022	OP850021|Italy|2022/09/12
OP850022*	Pica pica	Bird	Homogenate	Europe	Italy	Veneto	12/8/2022	OP850022|Italy|2022/08/12
OP850023*	Equus caballus	Equid	Brain	Europe	Italy	Campania	3/10/2022	OP850023|Italy|2022/10/03
MW835351*	Pica pica	Bird	Viral strain	Europe	Italy	Emilia-Romagna	2008	MW835351|Italy|2008/06/15
MW835352*	Equus asinus	Equid	Viral strain	Europe	Italy	Emilia-Romagna	13/9/2008	MW835352|Italy|2008/09/13
MW835353*	Equus caballus	Equid	Viral strain	Europe	Italy	Emilia-Romagna	8/9/2008	MW835353|Italy|2008/09/08
MW835354*	Equus caballus	Equid	Viral strain	Europe	Italy	Veneto	15/9/2008	MW835354|Italy|2008/09/15
MW835355*	Pica pica	Bird	Viral strain	Europe	Italy	Emilia-Romagna	31/7/2009	MW835355|Italy|2009/07/31
MW835356*	Pica pica	Bird	Viral strain	Europe	Italy	Emilia-Romagna	2011	MW835356|Italy|2011/06/15
MW835357*	Asio otus	Bird	Viral strain	Europe	Italy	Emilia-Romagna	2011	MW835357|Italy|2011/06/15
MW835358*	Corvus corone	Bird	Viral strain	Europe	Italy	Emilia-Romagna	2011	MW835358|Italy|2011/06/15
MW835359*	Gallus gallus	Bird	Viral strain	Europe	Italy	Sardinia	23/9/2011	MW835359|Italy|2011/09/23
MW835360*	Garrulus glandarius	Bird	Viral strain	Europe	Italy	Sardinia	19/9/2011	MW835360|Italy|2011/09/19
MW835361*	Equus caballus	Equid	Viral strain	Europe	Italy	Sardinia	15/9/2011	MW835361|Italy|2011/09/15
MW835362*	Athene noctua	Bird	Viral strain	Europe	Italy	Sardinia	25/9/2011	MW835362|Italy|2011/09/25
MW835363*	Gallus gallus	Bird	Viral strain	Europe	Italy	Sardinia	3/10/2011	MW835363|Italy|2011/10/03

MW835364*	Pica pica	Bird	Viral strain	Europe	Italy	Friuli-Venezia Giulia	20/12/2012	MW835364|Italy|2011/12/20
