# Supplementary material for: Origin and evolution of West Nile virus lineage 1 in Italy
Source: Epidemiol Infect. 2024 Dec 2;152:e150. doi: 10.1017/S0950268824001420 (PMC11626449; doi:10.1017/S0950268824001420)
Supplement: Silverj et al. supplementary material 5 — Silverj et al. supplementary material [file S0950268824001420sup005.pdf]

Accession	ID	Country	Region	Municipality	Host	Coordinate_Latitude	Coordinate_Longitude	Coordinate_Origin
HM152775	HM152775.1|Israel|2000/08/31	Israel	NA	Tel-Aviv	Human	32.08	34.78	NCBI-info
AY701412	AY701412.1|Morocco|1996/08/31	Morocco	NA	Rabat	Horse	34.03	-6.83	Inferred from municipality
MW835364	MW835364|Italy|2012/12/20	Italy	Friuli-Venezia Giulia	Livenza	Bird	45.68	13.09	NCBI-info
JX556213	JX556213.1|Italy|2012/08/03	Italy	Veneto	Venice	Human	45.44	12.34	NCBI-info
JQ928174	JQ928174.1|Italy|2011/09/15	Italy	Veneto	Treviso	Human	45.67	12.25	NCBI-info
KC954092	KC954092.1|Italy|2012/09/15	Italy	Veneto	Portogruaro	Human	45.78	12.83	NCBI-info
KF823807	KF823807.1|Italy|2013/09/15	Italy	Veneto	Padua	Human	45.41	11.87	NCBI-info
KF647253	KF647253.1|Italy|2013/08/15	Italy	Veneto	Rovigo	Human	45.07	11.79	NCBI-info
AY268132	AY268132.1|France|2000/08/31	France	NA	Camargue	Horse	43.62	4.53	Paper
JN858069	JN858069.1|Italy|2011/09/15	Italy	Marche	Ancona	Human	43.62	13.52	NCBI-info
JQ928175	JQ928175.1|Italy|2011/09/15	Italy	Veneto	Treviso	Human	45.67	12.25	NCBI-info
JF707789	JF707789.1|Spain|2008/08/31	Spain	Andalusia	Seville	Mosquito	37.39	-5.99	Paper
FJ766331	FJ766331.1|Spain|2007/10/15	Spain	NA	Madrid	Bird	40.42	-3.70	Inferred from municipality
FJ766332	FJ766332.1|Spain|2007/10/15	Spain	NA	Madrid	Bird	40.42	-3.70	Inferred from municipality
AF404757	AF404757.1|Italy|1998/09/03	Italy	Tuscany	Florence	Bird	43.80	10.81	Paper
AY701413	AY701413.1|Morocco|2003/08/31	Morocco	NA	Kenitra	Horse	34.30	-6.50	Paper
AJ965628	AJ965628.2|Portugal|2004/07/15	Portugal	Algarve	Almancil	Mosquito	37.07	-8.03	Paper
DQ786573	DQ786573.1|France|2004/10/21	France	NA	Camargue	Bird	43.62	4.53	Paper
DQ786572	DQ786572.1|France|2004/10/18	France	NA	Camargue	Bird	43.62	4.53	Paper
JF719069	JF719069.1|Spain|2010/08/15	Spain	Andalusia	Cadiz	Horse	36.54	-6.30	Paper

MW627239	MW627239.1|Italy|2020/10/01	Italy	Campania	Trentola-Ducenta	Bird	40.98	14.17	IZS-Teramo
JF719067	JF719067.1|Italy|2009/11/15	Italy	Emilia-Romagna	Ferrara	Bird	44.83	11.62	Paper
MW 835351	MW835351|Italy|2008/08/31	Italy	Emilia-Romagna	Ferrara	Bird	44.81	11.77	IZS-Teramo
JF719066	JF719066.1|Italy|2008/09/15	Italy	Emilia-Romagna	Ferrara	Bird	44.81	11.77	Paper
KF234080	KF234080.1|Italy|2009/08/31	Italy	Emilia-Romagna	Bologna	Human	44.49	11.34	Inferred from municipality
JF719068	JF719068.1|Italy|2009/10/15	Italy	Emilia-Romagna	Ferrara	Bird	44.81	11.77	Paper
GU011992	GU011992.2|Italy|2009/07/01	Italy	Veneto	Padua	Human	45.41	11.87	Inferred from municipality
MW835353	MW835353|Italy|2008/09/08	Italy	Emilia-Romagna	Ferrara	Horse	44.89	11.65	IZS-Teramo
FJ483548	FJ483548.1|Italy|2008/08/31	Italy	Emilia-Romagna	Bologna	Bird	44.49	11.34	Inferred from municipality
MW835352	MW835352|Italy|2008/09/13	Italy	Emilia-Romagna	Tresignana	Horse	44.80	11.89	IZS-Teramo
KU573077	KU573077.1|Italy|2008/08/31	Italy	Emilia-Romagna	Ferrara	Bird	44.81	11.77	Inferred from municipality
FJ483549	FJ483549.1|Italy|2008/08/31	Italy	Emilia-Romagna	Ferrara	Bird	44.81	11.77	Inferred from municipality
KU573078	KU573078.1|Italy|2009/08/31	Italy	Emilia-Romagna	Reggio-Emilia	Mosquito	44.70	10.63	Inferred from municipality
MW835355	MW835355|Italy|2009/07/31	Italy	Emilia-Romagna	Reggio-Emilia	Bird	44.70	10.63	IZS-Teramo
KU573079	KU573079.1|Italy|2009/08/31	Italy	Emilia-Romagna	Reggio-Emilia	Bird	44.70	10.63	Inferred from municipality
MW835354	MW835354|Italy|2008/09/15	Italy	Veneto	Trecenta	Horse	45.01	11.46	IZS-Teramo
JF719065	JF719065.1|Italy|2008/10/15	Italy	Emilia-Romagna	Modena	Bird	44.65	10.93	Paper
OU953896	OU953896.1|Spain|2020/09/09	Spain	Andalusia	Cadiz	Human	36.50	-6.34	Paper
OU953897	OU953897.1|Spain|2020/08/13	Spain	Andalusia	Seville	Human	37.39	-5.99	Paper
OU953895	OU953895.1|Spain|2020/08/13	Spain	Andalusia	Seville	Human	37.39	-5.99	Paper
MW835358	MW835358|Italy|2011/08/31	Italy	Emilia-Romagna	Bologna	Bird	44.49	11.34	IZS-Teramo
MW835362	MW835362|Italy|2011/09/25	Italy	Sardinia	Oristano	Bird	39.91	8.61	IZS-Teramo

MW835359	MW835359|Italy|2011/09/23	Italy	Sardinia	Santa Giusta	Bird	39.83	8.60	IZS-Teramo
MW835360	MW835360|Italy|2011/09/19	Italy	Sardinia	Oristano	Bird	39.78	8.68	IZS-Teramo
MW835361	MW835361|Italy|2011/09/15	Italy	Sardinia	Oristano	Horse	39.90	8.57	IZS-Teramo
MW835363	MW835363|Italy|2011/10/03	Italy	Sardinia	Arborea	Bird	39.72	8.53	IZS-Teramo
MW835357	MW835357|Italy|2011/08/31	Italy	Emilia-Romagna	Ferrara	Bird	44.81	11.77	IZS-Teramo
MW835356	MW835356|Italy|2011/08/31	Italy	Emilia-Romagna	Ferrara	Bird	44.81	11.77	IZS-Teramo
MT863559	MT863559.1|France|2015/10/03	France	NA	Camargue	Horse	43.62	4.53	Paper
OP009521	OP009521.1|Italy|2021/08/23	Italy	Veneto	Padua	Mosquito	45.41	11.87	NCBI-info
OP009523	OP009523.1|Italy|2021/08/16	Italy	Veneto	Padua	Mosquito	45.41	11.87	NCBI-info
OP009524	OP009524.1|Italy|2021/08/16	Italy	Veneto	Padua	Mosquito	45.41	11.87	NCBI-info
OP009522	OP009522.1|Italy|2021/08/16	Italy	Veneto	Padua	Mosquito	45.41	11.87	NCBI-info
OP009525	OP009525.1|Italy|2022/06/21	Italy	Veneto	Venice	Mosquito	45.44	12.34	NCBI-info
OP009520	OP009520.1|Italy|2021/10/11	Italy	Veneto	Padua	Bird	45.41	11.87	NCBI-info
OP734267	OP734267|Italy|2022/07/12	Italy	Emilia-Romagna	Mesola	Mosquito	44.92	12.18	IZS-Teramo
OP734274	OP734274|Italy|2022/07/14	Italy	Veneto	Villanova del Ghebbo	Mosquito	45.06	11.64	IZS-Teramo
OP734272	OP734272|Italy|2022/07/04	Italy	Veneto	Villanova del Ghebbo	Bird	45.07	11.66	IZS-Teramo
OP734271	OP734271|Italy|2022/07/13	Italy	Veneto	Padua	Bird	45.40	11.88	IZS-Teramo
OP734269	OP734269|Italy|2022/07/18	Italy	Veneto	Rovigo	Bird	45.07	11.79	IZS-Teramo
OP734270	OP734270|Italy|2022/07/19	Italy	Emilia-Romagna	Baricella	Mosquito	44.68	11.57	IZS-Teramo
OP734266	OP734266|Italy|2022/07/12	Italy	Emilia-Romagna	Riva del Po	Mosquito	44.98	11.95	IZS-Teramo
OP734268	OP734268|Italy|2022/07/07	Italy	Veneto	Rovigo	Mosquito	45.07	11.77	IZS-Teramo
OP734263	OP734263|Italy|2022/07/07	Italy	Veneto	Tribano	Mosquito	45.21	11.83	IZS-Teramo

OP734264	OP734264|Italy|2022/06/28	Italy	Veneto	Porto Tolle	Bird	44.92	12.42	IZS-Teramo
OP734265	OP734265|Italy|2022/07/07	Italy	Veneto	Rovigo	Bird	45.07	11.79	IZS-Teramo
OP734262	OP734262|Italy|2022/06/21	Italy	Veneto	Cavarzere	Mosquito	45.14	12.12	IZS-Teramo
OP850021	OP850021|Italy|2022/09/12	Italy	Lombardy	Sermide	Bird	44.98	11.27	IZS-Teramo
OP850022	OP850022|Italy|2022/08/12	Italy	Veneto	Zero Branco	Bird	45.59	12.15	IZS-Teramo
OP850023	OP850023|Italy|2022/10/03	Italy	Campania	Castel Volturno	Bird	41.01	13.98	IZS-Teramo
AJ965626.2 (Portugal) and OP734273 (Italy) discarded from the phylogeographic analysis after quality filtering								
