# Supplementary material for: Origin and evolution of West Nile virus lineage 1 in Italy
Source: Epidemiol Infect. 2024 Dec 2;152:e150. doi: 10.1017/S0950268824001420 (PMC11626449; doi:10.1017/S0950268824001420)
Supplement: Silverj et al. supplementary material 6 — Silverj et al. supplementary material [file S0950268824001420sup006.pdf]

NCBI Accession number	Sample number	Sequencing Methodology	#RUN	Genpat NRG Code	Total trimmed reads	WNV L1 reads	HCoverage	VCoverage	Consensus Length
OP734262	2022.TE.39540.1.1	Illumina	1	2022.TE.40159.2.3	3.495.685	744.562	99%	5560.68	10.968
-	Negative control	Illumina	1	2022.TE.4336.1.8	6	0	0%	0	0
OP734263	2022.TE.40956.1.1	Illumina	2	2022.TE.41224.2.8	7.571.826	597.499	98%	3973.2	10.967
OP734264	2022.TE.41166.1.1	Illumina	2	2022.TE.41224.2.16	14.648.025	1.333.187	100%	6381.67	11.016
OP734265	2022.TE.41302.1.1	Illumina	2	2022.TE.41400.2.5	11.441.518	1.107.291	99%	5888.49	10.961
OP734266	2022.TE.41296.1.1	Illumina	2	2022.TE.41400.2.3	4.662.340	220.508	91%	2381.21	10.963
OP734267	2022.TE.41297.1.1	Illumina	2	2022.TE.41400.2.4	6.865.408	269.727	94%	2618.84	10.962
OP734268	2022.TE.40958.1.1	Illumina	2	2022.TE.41224.2.10	4.560.711	283.771	93%	2899.47	10.960
-	Negative control	Illumina	2	2022.TE.4336.1.10	5	0	0%	0	0
OP734269	2022.TE.42122.1.1	Illumina	3	2022.TE.42275.2.20	6.447.209	657.948	99%	5135.45	10.966
OP734270	2022.TE.41754.2.1	Illumina	3	2022.TE.42275.2.11	3.371.541	369.610	96%	3287.31	10.956
OP734271	2022.TE.41727.1.1	Illumina	3	2022.TE.42275.2.10	5.382.363	629.364	99%	4806.11	10.960
OP734272	2022.TE.42106.1.1	Illumina	3	2022.TE.42275.2.18	3.942.375	304.215	97%	3084.4	10.962
OP734273*	2022.TE.41849.1.2	Illumina	3	2022.TE.42275.2.13	1.557.373	263.149	90%	2471.3	10.954
OP734274	2022.TE.41525.1.1	Illumina	3	2022.TE.42275.2.6	3.576.787	403.166	94%	3761.48	10.966

NCBI Accession number	Sample number	Sequencing Methodology	#RUN	Genpat NRG Code	Total trimmed reads	WNV L1 reads	HCoverage	VCoverage	Consensus Length
-	Negative control	Illumina	3	2022.TE.92.1.5	17.224	0	0%	0	0
OP850021	2022.TE.49076.2.10	Illumina	4	2022.TE.49076.2.10	2.347.380	351.292	99%	4177.69	10.963
OP850022	2022.TE.48124.2.3	Illumina	4	2022.TE.48124.2.3	2.923.237	387.986	99%	4264.56	10.966
OP850023	2022.TE.48124.2.1	Illumina	4	2022.TE.48124.2.1	1.805.957	159.703	92%	2101.31	10.960
-	Negative control	Illumina	4	2022.TE.92.1.3	468.755	0	0%	0	0
MW627239	2020.TE.238412	Illumina	5	2020.TE.362447.1.2	2.037.215	591.954	100%	6362.04	11.027
-	Negative control	Illumina	5	2020.TE.6002.1.1	5	0	0%	0	0
* OP734273: discarded from molecular and evolutionary analyses after quality filtering									

Accession	Sample number	Sequencing Methodology	Consensus Length
MW835351	2008.TE.15076	Sanger	10.252
MW835352	2008.TE.15242	Sanger	10.252
MW835353	2008.TE.15250	Sanger	10.252
MW835354	2008.TE.15325	Sanger	10.252
MW835355	2009.TE.12010	Sanger	10.302
MW835356	2011.TE.14444	Sanger	10.252
MW835357	2011.TE.17196	Sanger	10.252
MW835358	2011.TE.17208	Sanger	10.252
MW835359	2011.TE.20224_1	Sanger	10.252
MW835360	2011.TE.20875	Sanger	10.252
MW835361	2011.TE.21370	Sanger	10.252
MW835362	2011.TE.21412	Sanger	10.302
MW835363	2011.TE.23237_1	Sanger	10.252
MW835364	2012.TE.20652	Sanger	10.302

- ABI PRISM 3100 - Avant with POP7 polymer (Applied Biosystems)
- Assembling software = SeqScape v2.5 (Applied Biosystems)
- Sequence Reference = AF404757 Italy 1998 equine

Base calling:

- Use reference trimming
- Base spacing used = 15.55
- Base caller = KB.bcp
- Dyset/Primer = KB_3730_POP7_BDTv3.mob
- Quality value (QV) > 20
- Probability of error (Pe) < 1%
- Average coverage (per base) = 7x (each isolate was sequenced twice)
- Average sequence length = 700 nt
- Control DNA sequence = pGEM -3Zf (+) with the -21 M13 forward primer, included in BigDye Terminator v3.1 Cycle Sequencing Kit
