# Supplementary material for: Origin and evolution of West Nile virus lineage 1 in Italy
Source: Epidemiol Infect. 2024 Dec 2;152:e150. doi: 10.1017/S0950268824001420 (PMC11626449; doi:10.1017/S0950268824001420)
Supplement: Silverj et al. supplementary material 7 — Silverj et al. supplementary material [file S0950268824001420sup007.pdf]

Viral protein	Amino acid position	2021-22 North-Eastern Italian strains	OP850023 Italy/Campania/2022	MW627239 Italy/Campania/2020	2008 Italian strains	2011 Italian strains	AF404757 Italy/Tuscany/1998	MT863559 France/2015	OU953897 Spain/2020	JF719069 Spain/2010	AY701413 Morocco/2003	AJ965628 Portugal/2004	DQ786573 France/2004	NC_009942 USA/1999
C	S100L	S	S	S	S	S	S	S	S	S	S	S	L	S
preM	I35T	I	I	I	I	I	I	I	I	I	I	I	T	I
E	V24I	V	V	V	V	V	V	V	V	I	V	V	V	V
	A51T	A	T	T	A	A	A	A	A	A	A	A	A	A
	R93K	K	R	R	R	R	R	K	R	R	R	R	R	R
	T126I	T	T	T	T	T	T	T	T	T	T	T	T	I
	I159V	I	I	I	I	I	I	I	I	I	I	I	I	V
	F167L	F	F	F	F	F	F	F	F	F	F	F	F	L
NS1	A27V	A	A	A	A	A	A	A	A	A	A	V	A	A
	S70A	S	S	S	S	S	S	S	S	S	S	S	S	A
	P138S	P	P	P	P	P	P	P	P	P	P	P	S	P
	K141R	K	K	K	K	K	K	K	K	K	K	K	R	K
	I184V	I	V	I	I	I	I	I	I	I	I	I	I	I
	V188I	V	V	V	V	V	V	V	V	V	I	V	V	V
	L198M	L	M	L	L	L	L	L	L	L	L	L	L	L
	Q338R	Q	Q	Q	Q	Q	Q	Q	Q	R	Q	Q	Q	Q
	F17V	F	F	F	F	F	F	F	F	F	F	V	F	F
NS2A	I85V	I	I	I	V	I	I	I	I	I	I	I	I	I
	H119Y	H	H	H	H	H	H	H	H	H	Y	H	H	H
	R122H	H	R	R	R	R	R	R	R	R	R	R	R	R
	I158V	I	I	I	I	I	I	I	I	V	I	I	I	I
	G211R	G	G	G	G	G	G	G	R	G	G	G	G	G
	L159M	L	L	L	L	L	L	L	L	L	L	M	L	L
NS2B	M99T	M	T	T	M	M	M	M	M	M	M	M	M	M
NS3	F46L	F	F	F	F	F	F	F	F	F	L	F	F	F

	K142R	K	K	K	K	K	K	K	K	K	K	R	K	K
	I175V	I	I	I	I	I	I	I	I	I	I	V	I	I
	M239L	M	M	M	L	M	L	M	M	M	M	M	M	M
	Q244H	Q	Q	Q	Q	Q	Q	H	Q	Q	Q	Q	Q	Q
	T249P	P	T	T	P	T	T	T	T	T	T	P	T	P
	I356T	I	I	I	I	I	I	I	I	I	I	I	I	T
	T436A	T	T	T	T	A	T	T	A	T	T	T	T	T
	N465S	N	N	N	N	N	N	N	N	S	N	N	N	N
NS4A	V85I	V	V	V	I	V	V	V	V	V	V	V	V	A
	P100S	P	P	P	S	P	P	P	P	P	P	P	P	P
NS4B	S15R	S	S	S	S	S	S	R	S	S	S	S	S	S
	L96I	L	I	I	L	L	L	L	L	L	L	L	L	L
	D249E	D	D	D	D	D	D	D	D	D	D	D	D	E
NS5	R4K	K	K	K	K	K	K	R	K	K	K	K	K	K
	K41R	K	K	K	K	K	K	K	R	K	K	K	K	K
	K44R	R	R	R	R	R	R	K	R	R	R	R	R	R
	S237G	S	G	S	S	S	S	S	S	S	S	S	S	S
	V258A	V	V	V	A	A	V	V	V	V	V	V	V	V
	R422K	R	R	R	K	K	R	R	K	R	R	R	R	R
	K432R	R	R	R	K	K	K	K	K	K	K	K	K	R
